# Crosstalk Between Microbiome and Ferroptosis in Diseases: From Mechanism to Therapy

**DOI:** 10.1002/cph4.70042

**Published:** 2025-08-22

**Authors:** Si‐Qi Ding, Yun Lei, Zhe‐Ming Zhao, Xin‐Yun Li, Ji‐Xuan Lang, Jia‐Kui Zhang, Yong‐Shuang Li, Chun‐Dong Zhang, Dong‐Qiu Dai

**Affiliations:** ^1^ Department of Surgical Oncology The Fourth Affiliated Hospital of China Medical University Shenyang Liaoning China; ^2^ Central Laboratory The Fourth Affiliated Hospital of China Medical University Shenyang Liaoning China; ^3^ Cancer Center The Fourth Affiliated Hospital of China Medical University Shenyang China

**Keywords:** fecal microbiota transplantation, ferroptosis, gut‐organ‐axis, microbiome, probiotics

## Abstract

The human microbiome is a unique organ and maintains host immunomodulation and nutrient metabolism. Structural and functional microbiome alterations are commonly known as dysbiosis, which is strongly associated with disease progression. Ferroptosis is a novel iron‐dependent cell death mode characterized by intracellular iron accumulation, increased reactive oxygen species (ROS), and lipid peroxidation (LPO). Importantly, the complex crosstalk between the microbiome and ferroptosis in disease has attracted considerable research attention. The microbiome influences ferroptosis by regulating host iron homeostasis, mitochondrial metabolism, and LPO, among many other pathways. Thus, the in‐depth analysis of microbiome–ferroptosis crosstalk and associated mechanisms could provide new strategies to treat human diseases. Therefore, understanding this crosstalk is critical. Here, we systematically explore the associations between gut microbiome and ferroptosis across multiple diseases. We show that the oral microbiome also influences disease progression by regulating ferroptosis. Furthermore, we provide a potential for certain disease therapies by targeting the crosstalk between the microbiome and ferroptosis.

## Graphical abstract

The human microbiome, a key homeostatic hub, interacts with ferroptosis in diseases. This review dissects the crosstalk between the microbiome and ferroptosis, providing strategies for disease therapy. FMT, fecal microbiota transplantation; ROS, reactive oxygen species; TCM, traditional Chinese medicine.

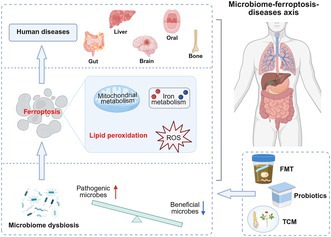

**Abbreviations**
AA‐PE: arachidonic acid‐phosphatidylethanolamine
ACSL4: acyl‐CoA synthetase long‐chain family member 4
AD: Alzheimer's disease
AHR: aryl hydrocarbon receptor
AIEC: adherent‐invasive *E. coli*

ALD: alcoholic liver disease
ALDH1A3: aldehyde dehydrogenase 1 family member A3
ALI: acute liver injury
APAP: acetaminophen
ATF3: transcription factor 3
Aβ: β‐amyloid
BAs: bile acids
CagA: cytotoxin‐associated gene A
CAT: capsiate
CD: Crohn's disease
CDCA: chenodeoxycholic acid
CNS: central nervous system
CoQ: ubiquinone
CoQH2: ubiquinol
CRC: colorectal cancer
DA: dopaminergic
DAMPs: danger‐associated molecular patterns
DCA: deoxycholic acid
DMT1: divalent metal transporter 1
DSS: dextran sodium sulfate
Ferritinophagy: ferritin autophagy
FMT: fecal microbiota transplant
FPN: ferroportin
FSP1: ferroptosis suppressor protein 1
FXR: farnesoid X receptor
Glu: glutamate
GLUT1: glucose transporter‐1
GPX4: glutathione peroxidase 4
GSH: glutathione
H_2_O_2_
: hydrogen peroxide
HCC: hepatocellular carcinoma
HDAC: histone deacetylase
HIF‐1α: hypoxia‐inducible factor‐1α
HIF‐2α: hypoxia‐inducible factor‐2α
IBD: inflammatory bowel disease
ICC: intrahepatic cholangiocarcinoma
IDA: trans‐3‐indoleacrylic acid
IEC: intestinal epithelial cell
IRI: ischemia reperfusion injury
IS: ischemic stroke
KC: Kupffer cells
KEAP1: Kelch‐associated protein 1
ALOX: lipoxygenase
LPCAT3: lysophosphatidylcholine acyltransferase 3
LPO: lipid peroxidation
LPS: lipopolysaccharide
MAFLD: metabolic dysfunction‐associated fatty liver disease
NAFLD: nonalcoholic fatty liver disease
NCOA4: nuclear receptor co‐activator 4
NOX2: NADPH oxidases 2
NOXs: NADPH oxidases
NRF2: nuclear factor erythroid 2‐related factor 2
OCA: obeticholic acid
OFG: oral fecal gavage
OPG: pasteurized fecal gavage
PD: Parkinson's disease
PDLSCs: periodontal ligament stem cells
POR: oxidoreductase cytochrome P450 reductase
PPAR: peroxisome proliferator‐activated receptor
PUFA: polyunsaturated fatty acid
PUFA‐ePLs: polyunsaturated ether phospholipids
PUFA‐PL: polyunsaturated fatty acid‐phospholipid
PUFA‐PL‐OOH: PUFA‐PL hydroperoxides
RA: rheumatoid arthritis
SCFAs: short‐chain fatty acids
SFXN1: siderofexin
SLC2A1: solute carrier family 2 member 1
SLC3A2: solute carrier family 3 member 2
SLC7A11: solute carrier family 7a member 11
SN: substantia nigra
SOD2: superoxide dismutase 2
TBI: total body irradiation
TCM: traditional Chinese medicine
TFR1: transferrin receptor 1
TLR4: toll‐like receptor‐4
TNF‐α: tumor necrosis factor α
UC: ulcerative colitis
α‐syn: α‐synuclein
ROS: reactive oxygen species

## 1 Introduction

Ferroptosis is a form of nonapoptotic cell death that depends on iron accumulation in cells (Dixon et al. 2012) and leads to increased toxic lipid peroxide reactive oxygen species (ROS) (Alves et al. 2025). The biochemical mechanisms underpinning ferroptosis involve lethal lipid peroxidation (LPO), ROS overload, altered cell metabolism, and imbalanced redox homeostasis (Dixon and Pratt 2023). Ferroptosis is induced by decreased glutathione peroxidase 4 (GPX4) activity, which leads to lipid peroxide accumulation. Ferroptosis sensitivity is closely associated with multiple biological processes, including iron (Anandhan et al. 2023), amino acid (Swanda et al. 2023), and polyunsaturated fatty acid (PUFA) metabolisms (Doll et al. 2017), mitochondrial function (Yamashita et al. 2024), glutathione (GSH) synthesis (Jiang et al. 2024), and phospholipid processing (Qiu, Zandkarimi, et al. 2024). Furthermore, ferroptosis is associated with multiple benign and malignant gut (Huang, Yang, et al. 2024), liver (Yu et al. 2020), brain (Wang, Liu, Zhang, et al. 2024), oral (Torres et al. 2024), and bone diseases (Feng et al. 2024).

As a unique organ, the human microbiome has been gradually revealed (Baquero and Nombela 2012). The ecological stability of a healthy human microbiome depends on many factors, such as the environment (Liu et al. 2025), the diet (Lin et al. 2025), medication (Daruka et al. 2025), genetics (Liu et al. 2025), gender (Dunham et al. 2024), age (Pasolli et al. 2019), and delivery mode (Zhou et al. 2023). Human microbiome composition and diversity may be altered by habitat environmental changes and may manifest as decreased beneficial symbionts and increased opportunistic pathogens, or dysbiosis (Qing et al. 2025). Microbiome dysbiosis also induces multiple human diseases (Ambat et al. 2024; Thibaut et al. 2025; Bosch et al. 2024; Kambara et al. 2024; Essex et al. 2024).

Iron is a key trace element that maintains normal human physiological activities and microbiome homeostasis (Pereira et al. 2024), whereas iron overload causes ferroptosis and dysbiosis (Gu et al. 2024). It was previously reported that dysbiosis induces ferroptosis in different diseases (He et al. 2023; Liu, Liu, et al. 2024; Fu et al. 2024; Zha et al. 2025; Wang, Wang, et al. 2023; Wang, Pan, et al. 2025). Furthermore, therapies targeting crosstalk between the microbiome and ferroptosis have been efficacious in several human diseases and attracted considerable research attention (Davenport et al. 2017; Sun et al. 2023). However, the specific interaction mechanisms between the microbiome and ferroptosis, as well as their impact on diseases, remain unclear. This review first systematically introduces the mechanisms of ferroptosis and the composition of the microbiome. Subsequently, we summarize the crosstalk between the microbiome and ferroptosis and its influence on diseases across various systems. Finally, we review the methods and advancements in targeting both the microbiome and ferroptosis for disease treatment.

## 2 Ferroptosis Mechanisms

### 2.1 Iron Metabolism Mechanisms

The balance of intracellular iron (Fe^2+^/Fe^3+^) concentration is a key link in maintaining cell function, involving the uptake, storage, utilization, and excretion of iron. Iron is involved in many biochemical processes, including oxygen transport, energy production, immune regulation, DNA synthesis, and antioxidant defense (Sun et al. 2023; Bogdan et al. 2016). Iron mainly comes from aging red blood cells, which are phagocytosed by macrophages, and also from dietary supplements absorbed by intestinal cells (Ahlqvist et al. 2015; von Siebenthal et al. 2023). Through the complex modulation of key molecules such as transferrin, divalent metal transporter 1 (DMT1), ferritin, and ferroportin (FPN), iron uptake, storage, and use are dynamically balanced (Galy et al. 2024). Dietary gut iron enters cells via DMT1 (Noordine et al. 2024), whereas absorbed iron is exported to the peripheral blood by FPN (Lim et al. 2018). In plasma, Fe^3+^ is tightly bound to transferrin for transport, which reduces free iron production and protects the iron from oxidation (Li, Li, et al. 2025). After reaching target cells, transferrin enters cells via the transferrin receptor 1 (TFR1) (Feng et al. 2020); after which, Fe^3+^ dissociates from transferrin and is converted to Fe^2+^ by the six‐transmembrane epithelial antigen of prostate 3 in DMT1 (Meng et al. 2022). Inside cells, iron is mainly stored by ferritin; however, when a cell is iron‐deficient, ferritin converts Fe^3+^ into Fe^2+^ via ferritin autophagy (ferritinophagy) (Zhou et al. 2022). When iron dissolves, FPN transports it outside the cell, where it is converted to Fe^3+^ by ceruloplasmin for further recycling (Fuqua et al. 2018). Critically, these iron metabolism and circulation processes are strictly modulated to ensure a balance exists between physiological requirements and toxicity.

Balanced iron metabolism is regulated by multiple factors, such as intracellular iron concentrations and partial oxygen pressure (Maniscalchi et al. 2024; Mastrogiannaki et al. 2009). When iron is deficient, anemia and other diseases may occur; however, iron overload aggravates oxidative stress in cells and eventually causes ferroptosis (Ru et al. 2024). In 2012, ferroptosis was first described as a form of cell death induced by the small molecule erastin, which was characterized by GSH depletion and phosphoperoxidase GPX4 inactivation (Dixon et al. 2012). Distinct from apoptosis, pyroptosis, and autophagy, ferroptosis is characterized by iron overload and unlimited LPO accumulation (Stockwell et al. 2017). Morphologically, ferroptotic cells exhibit necrosis‐like changes, including lost plasma membrane integrity, cytoplasm, and organelle swelling, but a normal nucleus (Dixon and Olzmann 2024). Structurally, ferroptosis alters mitochondrial structures, including mitochondrial condensation, reducing or missing cristae, increasing membrane density, and rupturing mitochondrial outer membranes (Zhang, Zhou, Gu, et al. 2024). Ferroptosis is highly complex and depends on an imbalance between oxidation and antioxidant systems, which involve several signal pathways and molecules (Tang et al. 2021; Conrad and Pratt 2019).

### 2.2 Ferroptosis Driver Pathways

#### 2.2.1 Iron Overload

Imbalanced iron homeostasis causes intracellular iron overload and ferroptosis. Specifically, Fe^3+^ enters cells and is converted to Fe^2+^ via TFR1 and DMT1 actions, while ferritinophagy increases Fe^2+^ levels (Chen, Zhang, et al. 2024). These reactive Fe^2+^ enter the labile iron pool (LIP) to maintain intracellular iron homeostasis (Camarena et al. 2021). Excessive Fe^2+^ catalyzes hydroxyl radical and lipid peroxide formation in a non‐enzymatic manner via Fenton reactions or lipid autoxidation (Li, Han, et al. 2025). These molecules (hydroxyl radicals and lipid peroxides) further promote LPO, ultimately leading to ferroptosis (Jinson et al. 2024). Also, free radicals may damage intracellular proteins, nucleic acids, and lipids and further promote ferroptosis. Notably, the FerroOrange fluorescent probe and ferrozine (detect intracellular Fe^2+^ levels) or Prussian blue staining (verifies tissue iron overload) were used to monitor host iron homeostasis, which helps the experimenters determine the relationship between iron metabolism and ferroptosis (Yang, Gao, et al. 2024; Huang et al. 2025). Although the precise mechanisms underpinning iron involvement in ferroptosis remain elusive, its role as a key medium for ROS production emphasizes its importance (Zhang, Li, Wang, et al. 2024; Co et al. 2024).

#### 2.2.2 Mitochondrial Metabolism

Most unstable intracellular iron is directly sent to the mitochondria to meet its normal functions (Das et al. 2016). However, excessive Fe^2+^ intake may increase ROS production via Fenton reactions, making the mitochondria the main ROS source in cells (Zheng et al. 2018; Willems et al. 2015). Among them, electrons are catalyzed via transfer by NADPH oxidases (NOXs) (Liu, Shi, et al. 2024) or leakage from electron transfer chain complexes I and III to produce superoxide, which is converted to hydrogen peroxide (H_2_O_2_) via superoxide dismutase 2 (SOD2) (Qiu, Sun, et al. 2024). H_2_O_2_ is further reduced by GPX4 (Schwarz et al. 2023) or it reacts with unstable iron via Fenton reactions to generate hydroxyl radicals (Illés et al. 2019). This promotes increased ROS levels and drives PUFA‐phospholipid (PUFA‐PL) peroxidation to induce ferroptosis (Zheng and Conrad 2020). Furthermore, free iron overload causes mitochondrial damage, which causes uncontrolled mitophagy, resulting in large quantities of free iron, ROS, and lipid peroxides entering the cytoplasm and exacerbating ferroptosis (Yu et al. 2022). When ferroptosis occurs, mitochondrial condensation and membrane rupture (distinct from apoptosis/necrosis) can be directly observed using transmission electron microscopy (Dixon et al. 2012). Therefore, limiting excess Fe^2+^ availability could strategically facilitate cellular antioxidant defenses.

#### 2.2.3 LPO

Lipid metabolism disorders are closely associated with ferroptosis (Feng and Stockwell 2018). LPO refers to the oxidation, fragmentation, and shortening of PUFAs or allyl‐rich phosphatidylethanolamine on lipid membranes by oxygen free radicals (Li, Hu, et al. 2024). This process generates cytotoxic substances such as lipid free radicals and active aldehydes, which cause cell disintegration and death by destroying cell lipid bilayers, proteins, and DNA structures (Yan et al. 2021). PUFA peroxidation via enzymatic reactions is the main cause of ferroptosis. Free PUFA reacts with CoA via acyl‐CoA synthetase long‐chain family member 4 (ACSL4) (Doll et al. 2017) to produce PUFA‐CoA, which generates PUFA‐PL via lysophosphatidylcholine acyltransferase 3 (LPCAT3) (Kagan et al. 2017). PUFA‐PL then produces high ROS levels via different enzymes such as oxidoreductase cytochrome P450 reductase (POR) (Xie et al. 2024) and lipoxygenases (ALOXs) (Zou, Li, et al. 2020), which cause LPO and promote ferroptosis. In this enzymatic reaction pathway, ACSL4 is the most thoroughly studied regulator and a vital initiator of ferroptosis (Mishima et al. 2025). The incorporation of PUFAs into phospholipids is a vital step in ferroptosis, and this process requires the joint participation of ACSL4 and LPCAT3 to determine ferroptosis sensitivity (Cui et al. 2023). Increased expression of ACSL4 and LPCAT3 facilitates the conversion of PUFAs to phospholipids. The iron‐containing enzyme ALOXs is a key target for intracellular Fe^2+^ to induce ferroptosis in a non‐enzymatic manner. Intracellular iron overload will increase the catalytic activity of ALOXs and trigger LPO (Shintoku et al. 2017). Furthermore, ALOXs are also associated with the p53‐mediated ferroptosis pathway that is independent of ACSL4 (Chu et al. 2019). Notably, NOXs also initiate membrane‐associated ROS production, which is another source of ROS, to mediate LPO (Poursaitidis et al. 2017; Reis et al. 2023). By inhibiting the expression of these enzymes, the levels of ROS and LPO can be effectively reduced, endowing cells with more resistance to ferroptosis (Doll et al. 2017). These findings suggest that pharmacological inhibition or gene knockout of these key enzymes may be a potential strategy to reduce the occurrence of ferroptosis. In addition, by using methods such as the DCFH‐DA fluorescent probe and C11‐BODIPY, researchers can assess intracellular ROS levels to evaluate the efficacy of measures targeting these enzymes (Dixon et al. 2012; Yang, Gao, et al. 2024).

### 2.3 Ferroptosis Suppressor Pathways

#### 2.3.1 The Xc^−^‐GSH‐GPX4 Axis

This axis was the first ferroptosis prevention system to be identified (Lei et al. 2022). Xc^−^ is a membrane sodium‐dependent amino acid antiporter, which exchanges glutamate (Glu) and cystine at a 1:1 ratio via solute carrier family 3 member 2 (SLC3A2) and solute carrier family 7A member 11 (SLC7A11), while reducing cystine to cysteine and promoting cysteine (raw material for GSH synthesis) transport to cells (Koppula et al. 2018; Liu et al. 2020). GPX4 is a lipid repair enzyme that converts and reduces reactive PUFA‐PL hydroperoxides (PUFA‐PL‐OOH) to non‐reactive and non‐lethal PUFA‐PL alcohols (Rodencal et al. 2024), while oxidizing reduced GSH to oxidized GSH to maintain a balanced intracellular free radical content and regulating ferroptosis (Zhang, Dai, et al. 2023). The use of imidazole ketone erastin, erastin, and RSL3 can pharmacologically remove cystine, deplete GSH, and inhibit GPX4, respectively, which can reduce the antioxidant capacity in cells and increase lipid ROS levels, ultimately leading to ferroptosis (Badgley et al. 2020; Yang et al. 2014).

#### 2.3.2 The FSP1‐CoQH_2_ System

The ferroptosis suppressor protein 1 (FSP1)‐ubiquinone (CoQ) axis was identified as a second endogenous mechanism that inhibited LPO and ferroptosis (Bersuker et al. 2019). FSP1 is localized to the plasma membrane and converts CoQ to its reduced form, ubiquinol (CoQH_2_), due to its NADH: ubiquinone oxidoreductase activity (Doll et al. 2019). CoQH_2_ acts as a lipid‐soluble antioxidant that prevents LPO and inhibits ferroptosis in cell membranes by reducing lipid radicals (Alves et al. 2025; Nakamura et al. 2023).

#### 2.3.3 Other Pathways

**FIGURE 1:**
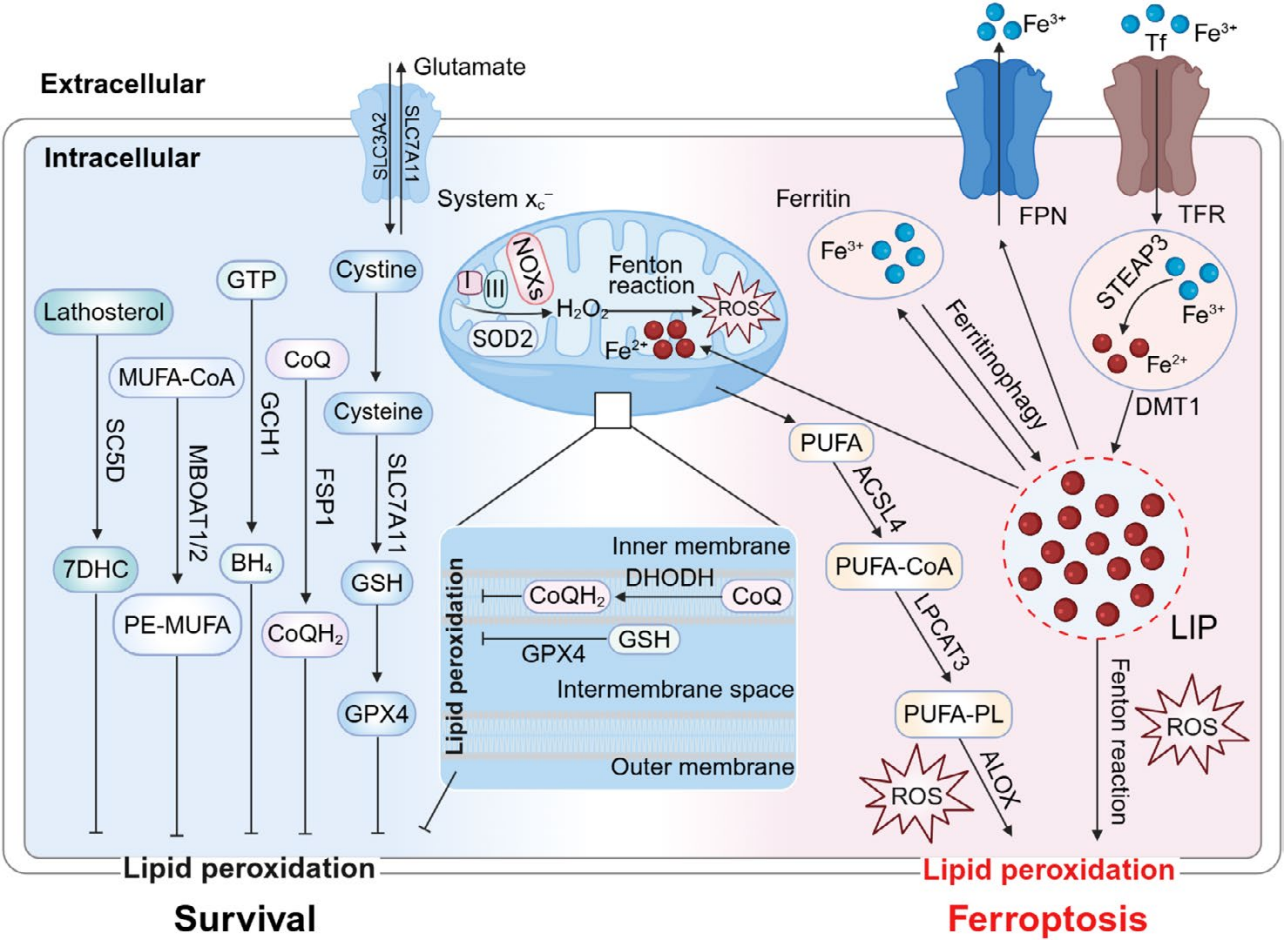
Ferroptosis driver or suppressor pathways. Ferroptosis driver pathways: TFR and DMT1 transport Fe^3+^ into cells and convert it to Fe^2+^. Intracellular iron is exported to extracellular sites by FPN. Fe^2+^ released by intracellular ferritinophagy promotes ROS production and lipid peroxidation via Fenton reactions. Most intracellular Fe^2+^ is used by the mitochondria. Electrons produce superoxide from electron transfer chain complexes I and III leak or NOXs transfer, which can be reduced to H_2_O_2_ by SOD2. H_2_O_2_ also reacts with unstable iron to produce abundant ROS (Fenton reactions). Additionally, free iron overload causes uncontrolled mitophagy, resulting in high free iron, ROS, and lipid peroxide levels entering the cytoplasm and exacerbating ferroptosis. PUFA is catalyzed by ACSL4 to produce PUFA‐CoA, which produces PUFA‐PL under LPCAT3. PUFA‐PL produces many ROS molecules through POR and ALOX actions, causing LPO and promoting ferroptosis. Ferroptosis suppressor pathways: Ferroptosis inhibition pathways include: Xc^−^‐GSH‐GPX4, FSP1‐CoQH_2_, DHODH‐CoQH_2_, GCH1‐BH4, MBOAT1/2‐MUFA, and SC5D‐7‐DH axis systems. SLC3A2 and SLC7A11 exchange glutamate and cystine, while reducing cystine to cysteine to synthesize GSH and promote GPX4 production. ACSL4, acyl‐CoA synthetase long‐chain family member 4; ALOX, lipoxygenase; CoQH_2_, ubiquinol; DMT1, divalent metal transporter 1; FPN, ferroportin; FSP1, ferroptosis suppressor protein 1; GPX4, glutathione peroxidase 4; GSH, glutathione; LPCAT3, lysophosphatidylcholine acyltransferase 3; LPO, lipid peroxidation; NOXs, NADPH oxidases; POR, oxidoreductase cytochrome P450 reductase; PUFA, polyunsaturated fatty acid; PUFA‐PL, polyunsaturated fatty acid‐phospholipid; ROS, reactive oxygen species; SLC3A2, solute carrier family 3 member 2; SLC7A11, solute carrier family 7a member 11; SOD2, superoxide dismutase 2; STEAP3, six‐transmembrane epithelial antigen of prostate 3.

Thanks to in‐depth ferroptosis analyses, ferroptosis prevention mechanisms have been increasingly unraveled. Apart from the Xc^−^‐GSH–GPX4 axis and the FSP1‐CoQH_2_ system, DHODH‐CoQH_2_ (Mao et al. 2021), GCH1‐BH4 (Kraft et al. 2020; Soula et al. 2020), MBOAT1/2‐MUFA (Liang et al. 2023), and the SC5D‐7‐DHC axis systems (Freitas et al. 2024; Li, Ran, et al. 2024) have been identified and characterized as novel ferroptosis prevention systems (Figure 1).

## 3 The Microbiome

### 3.1 General Properties

**FIGURE 2:**
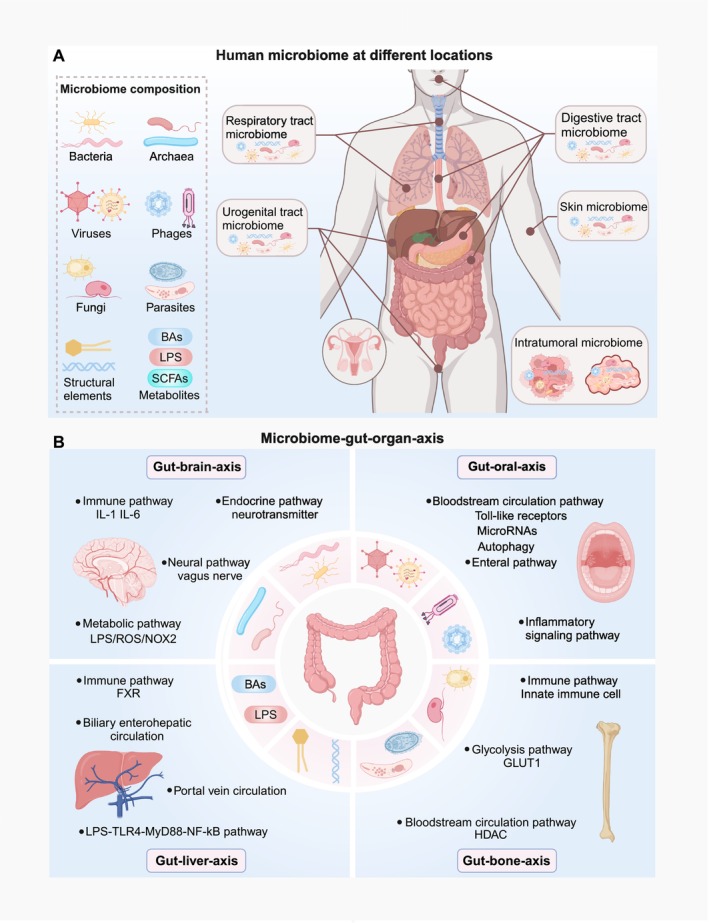
General microbiome properties and the microbiome‐gut‐organ axis. (A) The microbiome is distributed across different parts of the body, such as the digestive, respiratory, urogenital tract, and skin systems, and also tumor tissues. Microbiome composition includes bacteria, archaea, viruses, phages, fungi, and parasites, their structural elements, and various metabolites (e.g., LPS, SCFAs, and BAs). (B) The gut microbiome communicates with the liver, brain, oral system, and bone through immune, inflammatory, bloodstream, and other pathway systems to establish microbiome‐gut‐organ axes. BAs, bile acids; FXR, farnesoid X receptor; GLUT1, glucose transporter‐1; HDAC, histone deacetylase; LPS, lipopolysaccharide; NOX2, NADPH oxidases 2; SCFAs, short‐chain fatty acids; TLR4, toll‐like receptor‐4.

Broadly, the human microbiome consists of various microbiota systems, including not only microbiotas themselves (such as bacteria, archaea, fungi, viruses, phages, and small protists) but also their structural elements and various microbiota‐derived metabolites (such as toxins, amino acids, nucleic acids, lipopolysaccharide [LPS], short‐chain fatty acids [SCFAs], and bile acids [BAs]) (Berg et al. 2020; Glorieux et al. 2023; McCann and Rawls 2023; Carasso et al. 2024; Brown and Heneka 2024; Mann et al. 2024; Li, Ding, et al. 2024). The microbiome is distributed across different parts of the body, such as the digestive tract (oral, esophagus, and the gut), the respiratory tract (oral or nose, or the lung), the urogenital tract (kidney, vagina, uterus, and testes), the skin, and also various tumor tissues (Hou et al. 2022; Aggarwal et al. 2023), which maintain overall homeostasis by communicating within or between species and with the host (Manos 2022) (Figure 2A). Microbiome homeostasis is essential for human health, helping hosts to digest food and absorb nutrients, maintain intestinal barrier function, regulate the immune system, promote metabolism, and perform other assorted functions (Bolte et al. 2025; Zhao et al. 2023). Therefore, when dysbiosis occurs, diseases can arise (Aggarwal et al. 2023).

### 3.2 The Microbiome–Gut–Organ Axis

Gut microbiome homeostasis influences multiple human diseases (Lee et al. 2022). Such may be related to the complex interaction between the gut and other remote organs, called the gut‐organ axis (Zhang, Wang, Sang, et al. 2024; Kim and Sung 2024). Thanks to the communication ability of the microbiome, these axes are also known as microbiome‐gut‐organ axes, which are critical for human microbiome and host homeostasis (Hsu and Schnabl 2023; He et al. 2025; Baker et al. 2024; Zaiss et al. 2019). Here, we describe microbiome‐gut‐liver/brain/oral/bone axes, which have been relatively well‐studied in ferroptosis (Figure 2B).

#### 3.2.1 The Microbiome–Gut–Liver Axis

This axis depends on biliary enterohepatic circulation, portal vein circulation, and systemic mediators (Hsu and Schnabl 2023). Enterohepatic BA circulation is a major communication route between the liver and the gut. Specifically, BAs and other metabolites synthesized by the liver are released through the biliary tract into the gut and influence gut microbiome composition and intestinal barrier function (Tripathi et al. 2018). Gut BAs effectively inhibit microbial overgrowth by disrupting bacterial cell membranes (Kurdi et al. 2006) or binding to farnesoid X receptor (FXR) (Inagaki et al. 2006). Moreover, oral BAs reduce bacterial translocation and endotoxemia (Lorenzo‐Zúñiga et al. 2003), which may be related to the protective effects of BA‐mediated FXR activation on intestinal barrier integrity (Li, Chaudhari, et al. 2022). Gadaleta et al. reported that FXR activation reduced goblet cell loss, decreased intestinal permeability, and alleviated intestinal inflammation to improve inflammatory bowel disease (IBD) prognosis (Gadaleta et al. 2011).

The gut microbiome can be translocated to the liver via reabsorbed BAs or increased intestinal permeability, thereby inducing liver inflammation and damage (Tilg et al. 2022). Studies have confirmed that patients with liver diseases experience intestinal inflammation and gut microbiome dysbiosis (Boursier et al. 2016), which are inducible factors for increased intestinal permeability (Kang et al. 2022). Increased intestinal permeability causes microbes and microbial metabolites (e.g., LPS) to translocate to the liver through portal vein circulation (Pabst et al. 2023). Microbial metabolites also influence liver disease progression by regulating liver immune responses via FXR (Wen et al. 2024). LPS and acetaldehyde (ethanol metabolites) are recognized by immune receptors on Kupffer cells (KCs) and induce these cells to release pro‐inflammatory cytokines and chemokines, leading to host immune responses (Yan et al. 2011; Remetic et al. 2022). Also, commensal‐derived D‐lactate enhances clearance in KCs and helps these cells to establish an intravascular immune firewall to prevent pathogen transmission from the bloodstream (McDonald et al. 2020). Moreover, LPS accumulation in the liver induces myeloid differentiation factor‐88‐mediated nuclear factor‐kappa B activation via toll‐like receptor‐4 (TLR4) activation. These actions release inflammatory cytokines (Nighot et al. 2017), trigger liver inflammation and damage, and further deteriorate liver disease (Luther et al. 2015). Notably, probiotic supplementation can restore BAs and SCFAs, which reduces intestinal epithelial damage (Li, Wang, et al. 2022), regulates tight junctions (Zheng et al. 2017), and improves the intestinal barrier to reduce blood endotoxin levels and inhibit liver disease progression (Yu et al. 2021).

#### 3.2.2 The Microbiome–Gut–Brain Axis

Bidirectional communications between the gut and the brain are known as the microbiome‐gut‐brain axis, with the microbiome having key roles in this process (He et al. 2025). The gut microbiome affects physiological and pathological brain mechanisms mainly through metabolic, endocrine, neural, and immune pathways (Shandilya et al. 2022) Physiologically, the gut microbiome regulates the vagus nerve and several cytokines (e.g., interleukin (IL)‐1 and IL‐6) (Powell et al. 2017) by producing neurotransmitters and various metabolites (e.g., LPS and SCFAs) to regulate host immunity and neurodevelopment (Wang, Yang, and Liu 2023). The pathological regulation of the gut microbiome on the central nervous system (CNS) is mainly mediated by microglia (Loh et al. 2024), which, with either pro‐inflammatory M1 or anti‐inflammatory M2 phenotypes, are resident macrophages in the CNS (Yu, Chang, et al. 2023). It was previously reported that LPS crossed the intestinal barrier into the systemic circulation and across the blood–brain barrier, promoting microglia M1 polarization, which then released abundant pro‐inflammatory cytokines (e.g., tumor necrosis factor (TNF)‐α, IL‐1, and IL‐6), exacerbating neuroinflammation and neurodegenerative processes (Guo et al. 2022). Also, LPS‐induced macrophage ROS production and NADPH oxidase 2 (NOX2) activation were shown to further increase brain oxidative stress and damage (Wu et al. 2020). Yang, Hao, et al. (2023) observed that inhibited LPS‐induced NOX2 microglia activation effectively reduced oxidative stress and neuroinflammation caused by ischemia–reperfusion brain damage. In contrast, SCFAs promoted microglia polarization to M2 phenotypes, which facilitated anti‐inflammatory cytokine release (e.g., IL‐4 and IL‐13) (Ahuja and Lazar 2021) to inhibit neuroinflammation (Qian et al. 2022). Notably, reduced SCFA and increased LPS levels may cause α‐synuclein (α‐syn) aggregation, which increases ROS and LPO in dopaminergic (DA) neurons and also microglia activation and M1 polarization. This process was shown to generate more ROS and inflammatory cytokine levels and exacerbate neuroinflammation and neuronal injury (Kalyanaraman et al. 2024; Fang et al. 2024).

#### 3.2.3 The Microbiome–Gut–Oral Axis

The intimate relationship between the oral and gut microbiome supports the notion of a microbiome‐oral‐gut axis (Derrien et al. 2010; Chen, Peng, et al. 2024). Liao et al. (2024) reported that gut microbiome depletion increased relative oral microbiome abundance in the gut. Furthermore, oral inflammation and mucosal damage caused by periodontal disease allowed oral pathogens and their metabolites to enter the bloodstream (Sedghi et al. 2021), which potentially allowed the oral microbiome to participate in gut or systemic diseases (Baker et al. 2024). The oral pathogen *F. nucleatum* is a key pathogenic factor and colorectal cancer (CRC) biomarker (Wang and Fang 2023). The evidence now suggests that *F. nucleatum* is mainly colonized in the gut via an enteral route and promotes CRC and chemotherapy resistance by regulating TLRs, microRNAs, and inflammatory signaling pathways (Wang and Fang 2023; Yu et al. 2017; Zhang, Zhang, et al. 2022). Interestingly, *F. nucleatum* colonization can also be detected in CRC tissues by intravenously injected *F. nucleatum* , indicating that it can also be translocated to the gut through the bloodstream circulation (Abed et al. 2020). In contrast, gut microbes rarely colonize oral spaces and only appear in special cases, such as poor hygiene or hypoimmunity (Liu, Su, et al. 2022).

Notably, the links between the oral microbiome and liver diseases may be mediated by a novel oral‐gut‐liver axis (Albuquerque‐Souza and Sahingur 2022). *Porphyromonas gingivalis* ( *P. gingivalis* ) infection was reported as a risk factor for alcoholic liver disease (ALD) (Gao et al. 2023). Specifically, *P. gingivalis* , which enters the liver by inducing intestinal microbiome dysbiosis, was shown to aggravate liver inflammation in ALD mice via TLR4/IL‐6/TNF‐α/TGF‐β1 signaling (Gao et al. 2023). Moreover, *P. gingivalis* directly affects nonalcoholic fatty liver disease (NAFLD) progression via the circulation. *P. gingivalis* ‐associated LPS also promoted TLR2 and pro‐inflammatory cytokine expression in steatotic hepatocytes, leading to intracellular lipid accumulation and insulin resistance, which worsened NAFLD (Kim et al. 2024).

#### 3.2.4 The Microbiome–Gut–Bone Axis

The establishment of the microbiome‐gut‐bone axis depends on the regulation of the gut microbiome to remote organs through the bloodstream circulation (Zaiss et al. 2019). Previous studies reported that the gut microbiome could maintain bone homeostasis by regulating host immunity and metabolism (Guo et al. 2023; Lin et al. 2023), whereas, during dysbiosis, the gut microbiome caused bone disease by changing intestinal permeability (Zhang et al. 2021). Gut metabolites that reach the bone via the systemic circulation can impact bone metabolism through multiple mechanisms. For example, glucose transporter 1 (GLUT1) is mainly responsible for glucose transport in osteoblasts and provides materials for osteoblast glycolysis (Lee et al. 2017). A recent study reported that gut metabolites may regulate GLUT1 expression to regulate bone energy metabolism, thus influencing bone health (Guan et al. 2023). Furthermore, butyrate has important roles in bone diseases, as it inhibits histone deacetylase (HDAC) to stimulate osteoblast or anti‐osteoclast differentiation (Perego et al. 2018).

## 4 The Microbiome and Ferroptosis

The impact of the gut microbiome on ferroptosis in disease has gained considerable research traction (Yao and Li 2023; Liu, Wang, et al. 2024). Pathogenic bacteria usually exhibit promoting ferroptosis, of which mechanisms include promoting pro‐inflammatory cytokine secretion, labile iron accumulation, mitochondrial dysfunction, ROS production, and LPO. *Escherichia coli*‐derived LPS promotes IL‐6 secretion to increase the level of intracellular oxidative stress and Fe^2+^, thereby triggering ROS‐mediated ferroptosis in goat mammary epithelial cells (Zhu et al. 2022). While probiotics can enhance the levels of GSH and antioxidant enzymes by modulating iron absorption, generating SCFAs, and improving intestinal barrier function, they reduce oxidative stress and LPO and inhibit ferroptosis. *Lactobacillus*‐produced SCFAs enhance GSH levels, thereby suppressing ferroptosis in ischemia reperfusion injury (IRI) mice (Wang, Wang, et al. 2025). Notably, the oral microbiome can also influence multiple diseases by directly regulating ferroptosis.

### 4.1 Iron Homeostasis

Iron homeostasis is maintained by complex iron metabolic activities (uptake, storage, and utilization) (Galy et al. 2024). An imbalanced iron metabolism causes several health issues, such as iron deficiency‐related anemia (Pasricha et al. 2021) due to iron deficiency or ferroptosis (Yang et al. 2020) due to iron overload. Host iron metabolism affects microbiome diversity, distribution, and function. Because intestinal iron absorption (dietary iron or iron agents) is the sole external exogenous iron source in a physiological situation, controlling intestinal iron absorption is key to maintaining iron homeostasis (Bao et al. 2024). *Lactobacillus* species are reportedly implicated in intestinal iron levels, while their relative abundance decreases with iron overload (Shah et al. 2019) but increases with iron deficiency (Das et al. 2020). Several clinical trial studies have demonstrated that probiotics (such as *Lactobacillus*) and prebiotics can enhance intestinal iron absorption and alleviate intestinal inflammation (Hoppe et al. 2015; Mikulic et al. 2024). Furthermore, the microbiome regulates ferroptosis by regulating iron homeostasis in several ways (Xiao et al. 2023; Teschke 2024).

Siderophores are a type of metabolite with a high affinity for iron. Studies have shown that microbes indirectly regulate iron metabolism and ferroptosis by secreting siderophores to compete with the host for iron absorption (Schalk 2025). For instance, the siderophore pyoverdine secreted by *Pseudomonas aeruginosa* significantly reduces the intracellular iron concentration by chelating iron (Fe^2+^/Fe^3+^), thereby inhibiting ferroptosis in tumor cells (Yeung et al. 2024).

**FIGURE 3:**
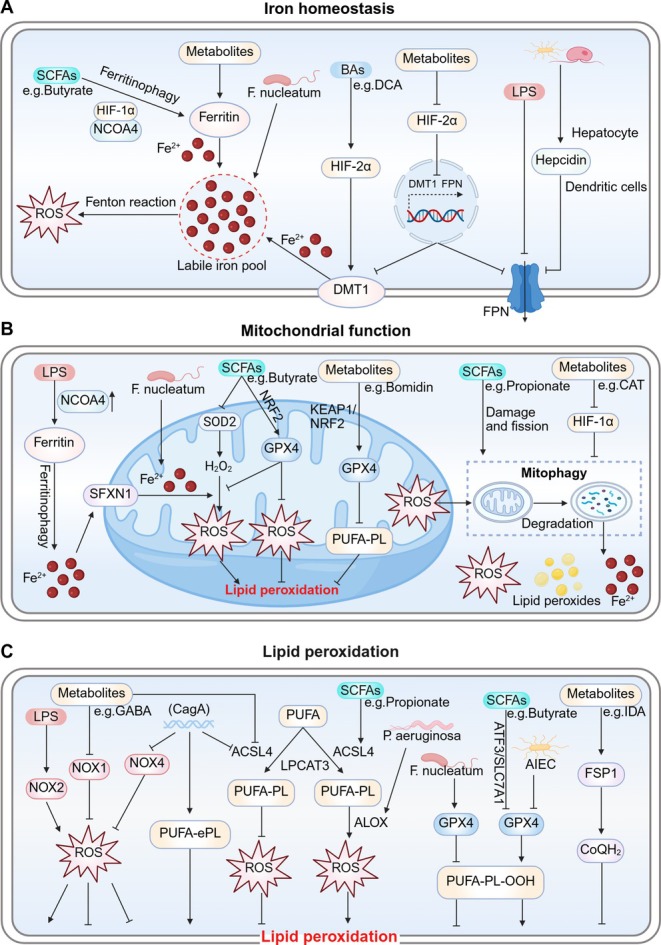
Microbial regulation of ferroptosis. (A) The microbiome inhibits FPN expression by inducing hepcidin or inhibiting HIF‐2α expression, and inducing intracellular iron overload. DCA promotes DMT1 expression via HIF‐2α, leading to increased labile iron levels. Microbial metabolites promote ferritin storage. Butyrate promotes HIF‐1α‐NCOA4‐mediated ferritinophagy, resulting in high Fe^2+^ levels entering the LIP and producing ROS, thereby inducing ferroptosis. *F. nucleatum* induces ferroptosis by increasing unstable iron levels in cells. (B) LPS induces NCOA4‐mediated ferritinophagy to activate SFXN1, and transfers excess Fe^2+^ to mitochondria, resulting in ferroptosis. *F. nucleatum* ‐induced iron overload may cause mitochondrial dysfunction. Excess iron accumulation in the mitochondria leads to the ROS, LPO, and mitophagy‐mediated aggravation of intracellular labile iron, which further induces ferroptosis. Additionally, propionate promotes mitochondrial damage, fission, and mitophagy. CAT also inhibits mitophagy and oxidative stress by inhibiting HIF‐1α expression to ultimately inhibit ferroptosis. Bomidin and butyrate increase GPX4 expression by upregulating the KEAP1/NRF2 pathway, which reduces mitochondrial damage and subsequent LPO. Tumoral butyrate increases intracellular ROS levels and mitochondrial metabolic damage by decreasing SOD2 expression. (C) Propionate elevates PUFA‐PL levels by increasing ACSL4 expression, while CagA reduces ACSL4 levels. CagA promotes PUFA‐ePLs synthesis, causing ferroptosis sensitivity in cells. *P. aeruginosa* promotes lipid oxidation by enhancing ALOX expression to induce ferroptosis. LPS‐induced NOX2 activation increases oxidative stress. Nevertheless, CagA or glutamine reduces NOX4 or NOX1 expression, respectively, both of which decrease ROS production and inhibit ferroptosis. AIEC colonization may decrease GPX4 expression, thus aggravating LPO and ferroptosis. *F. nucleatum* inhibits ferroptosis by elevating GPX4. Butyrate depletes intracellular GPX4 by inhibiting the ATF3/SLC7A11 axis to promote lipid peroxidation and ferroptosis. IDA increases FSP1‐CoQH_2_ levels and contributes to ferroptosis resistance. ACSL4, acyl‐CoA synthetase long‐chain family member 4; AIEC, adherent‐invasive *E. coli* ; ALOX, lipoxygenase; ATF3, transcription factor 3; CagA, cytotoxin‐associated gene A; CAT, Capsiate; CoQH2, ubiquinol; DCA, deoxycholic acid; DMT1, divalent metal transporter 1; FPN, ferroportin; FSP1, ferroptosis suppressor protein 1; GPX4, glutathione peroxidase 4; HIF‐1α, hypoxia‐inducible factor‐1α; HIF‐2α, hypoxia‐inducible factor‐2α; IDA, trans‐3‐indoleacrylic acid; KEAP1, Kelch‐associated protein 1; LIP, labile iron pool; LPO, lipid peroxidation; LPS, lipopolysaccharide; NCOA4, nuclear receptor co‐activator 4; NOX1/2/4, NADPH oxidases 1/2/4; NRF2, nuclear factor erythroid 2‐related factor 2; *P. aeruginosa* , *Pseudomonas aeruginosa* ; PUFA‐ePLs, polyunsaturated ether phospholipids; PUFA‐PL, polyunsaturated fatty acid‐phospholipid; SFXN1, siderofexin; SOD2, superoxide dismutase 2.

Hepcidin is a primary iron homeostasis regulator and limits iron release from cells by inducing FPN degradation, thus facilitating cellular iron overload (Taylor et al. 2011). While the microbiome induces hepcidin expression (hepatocyte‐derived or conventional dendritic cell‐derived) (Shanmugam et al. 2015; Bessman et al. 2020), there is no direct evidence that it regulates ferroptosis via hepcidin. What is known is that FPN expression is significantly reduced in LPS‐induced endotoxemia in rat atrial tissues, resulting in intracellular iron overload and triggering ferroptosis by increasing atrial oxidative stress (Fang et al. 2021). Notably, intestinal iron absorption by the hepcidin/FPN axis is mediated by hypoxia‐inducible factor‐2α (HIF‐2α) (Schwartz et al. 2019), which may facilitate intestinal iron absorption by modulating DMT1 and FPN (Taylor et al. 2011). Das et al. (2020) reported that in intestinal iron deficiency, elevated microbial metabolites regulated host iron homeostasis by inhibiting HIF‐2α activity, which limited intestinal iron absorption and upregulated ferritin expression to increase cellular iron storage. Another gut bacterial metabolite, deoxycholic acid (DCA), was reported to up‐regulate HIF‐2α and DMT1 expression, resulting in intracellular Fe^2+^ overload, which produced excessive ROS (Fenton reactions) and triggered oxidative stress, thus causing intestinal epithelial cell (IEC) ferroptosis (Wang, Chu, Dong, et al. 2024). In contrast, hypoxia‐inducible factor‐1α (HIF‐1α) is generally considered a ferroptosis inhibitor (Shen et al. 2024); however, HIF‐1α mediates ferritinophagy in promoting ferroptosis (Zhao et al. 2020). Nuclear receptor co‐activator 4 (NCOA4)‐dependent ferritinophagy is another regulatory process for maintaining intracellular and systemic iron homeostasis (Galy et al. 2024; Santana‐Codina et al. 2021). Butyrate secreted by oral pathogens, such as *P. gingivalis* , *Tannerella forsythia* , and *Prevotella intermedia* , promoted NCOA4‐mediated ferritinophagy, which was dependent on HIF‐1α, resulting in high Fe^2+^ quantities entering the transient LIP and producing ROS via Fenton reactions, thereby inducing ferroptosis (Zhao et al. 2020). Similarly, *F. nucleatum* ‐induced ferroptosis in periodontal ligament stem cells (PDLSCs) by increasing unstable iron levels (Wang, Wang, et al. 2023) (Figure 3A).

### 4.2 Mitochondrial Functions

As an intermediate link between iron overload and LPO, mitochondrial dysfunction is implicated in ferroptosis (Lei et al. 2022). Mitochondria are the main ROS source in the cell, and the microbiome regulates ferroptosis by regulating mitochondrial function. LPS‐induced NCOA4 overexpression induces the ferritinophagy‐mediated release of high Fe^2+^ quantities into the cytoplasm, resulting in siderofexin (SFXN1) activation on outer mitochondrial membranes (Li et al. 2020). Subsequently, the transfer of excess Fe^2+^ to mitochondria through SFXN1 is a self‐protection mechanism of cells in the early stage of iron overload (Zhang, Xin, et al. 2022). However, excess iron accumulation in the mitochondria leads to excess ROS and LPO, as well as extensive mitophagy, at the cost of aggravating intracellular unstable iron and further inducing ferroptosis (Chen et al. 2020; Li et al. 2023). For instance, *F. nucleatum* ‐induced iron overload leads to mitochondrial dysfunction, which induces ferroptosis in PDLSCs (Wang, Wang, et al. 2023). Propionate derived from the gut microbiome also promotes acute myeloid leukemia (AML) cell ferroptosis by inducing mitochondrial damage, fission, and mitophagy (Wei, Liu, et al. 2024). Under hypoxic conditions, HIF‐1 promoted mitophagy, resulting in increased oxidative stress and accelerated LPO to amplify ferroptosis (Li et al. 2023). Additionally, the gut metabolite capsiate (CAT) inhibits mitophagy and oxidative stress by inhibiting HIF‐1α expression, ultimately inhibiting ferroptosis in knee osteoarthritis mice (Guan et al. 2023).

Furthermore, the microbiome regulates ferroptosis by influencing mitochondrial antioxidant proteins. The antioxidant nuclear factor erythroid 2‐related factor 2 (NRF2), which is inhibited by kelch‐associated protein 1 (KEAP1) transcription (Bonkhoff et al. 2022), induces antioxidant proteins such as SOD2 and GPX4, which are cell‐protection defenses against ferroptosis (Gan 2021). During oxidative stress, NRF2 dissociates from KEAP1 to trigger downstream antioxidant protein transcription, which is a self‐protection mechanism in cells (Dodson et al. 2019). The *Escherichia coli* metabolite bomidin increases Xc^−^/GPX4 axis expression by upregulating KEAP1/NRF2 signaling, thus exerting anti‐ferroptosis and anti‐inflammatory effects in TNF‐α‐induced PDLSCs (Wu et al. 2024). H_2_O_2_ reduction by SOD2 and GPX4 cuts off mitochondrial PUFA‐PL peroxidation to reduce oxidative stress and maintain mitochondrial homeostasis (Kenny et al. 2019; Brigelius‐Flohé and Maiorino 2013). Gut butyrate was also shown to inhibit ferroptosis by inducing NRF2/GPX4 to reduce mitochondrial damage and subsequent LPO in colitis mice (Chen, Qian, et al. 2024). Additionally, the tumoral butyrate‐producing *Clostridium butyricum* enhances oxidative stress and mitochondrial metabolic damage in pancreatic ductal adenocarcinoma cells by decreasing SOD2 expression and significantly increasing intracellular ROS levels (Yang, Zhang, et al. 2023). These observations suggest that butyrate may have opposite roles at different sites via different mechanisms (Figure 3B). This may be related to the intricate tumor microenvironment and the cooperation between the host and microbiome.

### 4.3 LPO

Uncontrollable LPO is a critical hallmark of ferroptosis. The evidence now suggests that the microbiome influences ferroptosis by regulating LPO. PUFA‐PL susceptibility to LPO is due to its bis‐allylic hydrogen atom structure and ACSL4 catalysis (Conrad and Pratt 2019). Therefore, regulating ACSL4 expression can be an effective strategy to influence ferroptosis. Wei, Liu, et al. (2024) investigated the effects of different propionate concentrations on the progression of AML in vivo or in vitro, including oral administration (200 mM), intravenous injection (5 mg/100 μL/per mouse), and cell culture (0, 2.5, 5.0, 7.5, 10 mM). They found that different dosages of propionate induced AML cell ferroptosis by upregulating ACSL4 expression. Additionally, *Helicobacter pylori* cytotoxin‐associated gene A (CagA) (Lu et al. 2024) reduces ACSL4 levels, with potential anti‐ferroptosis roles. However, CagA was also shown to promote polyunsaturated ether phospholipid (PUFA‐ePL) synthesis, endowing gastric cancer cells with ferroptosis sensitivity (Peng, Lei, et al. 2024). This susceptibility was related to PUFA‐ePLs serving as additional substrates for LPO (Zou, Henry, et al. 2020). Unfortunately, there is little evidence on the impact of CagA on ferroptosis. Excess ROS produced by PUFA‐PL peroxidation is mediated by ALOX. The evidence now suggests that *P. aeruginosa* promotes LPO by enhancing ALOX expression to induce ferroptosis in human bronchial epithelial cells and mice IECs and shows how the microbiome affects ferroptosis‐associated iron‐containing enzymes (Dar et al. 2018, 2022). Additionally, NOX enzymes, which drive membrane‐associated ROS, are reportedly regulated by the microbiome. LPS‐induced NOX2 activation increased oxidative stress (Wu et al. 2020). Nevertheless, CagA overexpression or supplementation with glutamine reduced NOX4 or NOX1 expression, respectively, both of which significantly decreased ROS production and exerted ferroptosis inhibitory effects (Lu et al. 2024; Zhang, Zhou, Zhai, et al. 2024).

The microbiome‐mediated regulation of ferroptosis suppressor pathways is another important factor affecting lipid peroxide accumulation and ferroptosis. GPX4 is a well‐known ferroptosis defender with key roles in defending against LPO by hydrolyzing PUFA‐PL‐OOH (Seibt et al. 2019). Adherent‐invasive *Escherichia coli* (AIEC) cell colonization potentially decreased GPX4 expression; thus aggravating LPO and ferroptosis (Wen et al. 2023). In contrast, the gut microbial metabolite vinyl‐ether phospholipid (plasmalogen) inhibited ferroptosis by increasing GPX4 expression (Liu et al. 2023); unlike the previous PUFA‐ePLs, the absence of PUFA results in its insensitivity to H_2_O_2_ (Vítová et al. 2021). Interestingly, *F. nucleatum* also inhibited ferroptosis in CRC cells and a xenograft mouse model by elevating GPX4 levels, which were associated with GSH accumulation (Li, Wei, et al. 2024). GSH is an indispensable cofactor for correct GPX4 function, and its depletion inactivated GPX4 (Yang et al. 2014). Moreover, GSH synthesis was limited by cysteine availability, which depended on the Xc^−^ (SLC7A11) system. Cystine removal or interceding with SLC7A11‐mediated cystine transport (e.g., using the ferroptosis inducer erastin) also promoted ferroptosis (Koppula et al. 2021). Bi et al. (2024) reported that butyrate depleted intracellular GSH levels by inhibiting SLC7A11 expression via transcription factor 3 (ATF3) (Wang et al. 2020), which was possibly related to the HDAC inhibition of butyrate (Duncan et al. 2020), thereby promoting LPO and ferroptosis in lung cancer cells.

Notably, when GPX4 was inactivated, anti‐ferroptosis actions were mainly mediated by FSP1 (Doll et al. 2019), which in turn inhibited lipid peroxides by producing CoQH_2_, trapping lipid radicals, and thus inhibiting ferroptosis in various cell membrane structures (Bersuker et al. 2019). Previous studies have confirmed that several tryptophan metabolites, such as indole‐3‐pyruvate, serotonin, and 3‐hydroxyo‐aminobenzoic acid, can counteract ferroptosis by scavenging oxygen free radicals or activating NRF2/GSH synthesis (Zheng and Conrad 2025). In addition, microbial tryptophan metabolites have exhibited great potential in protecting intestinal barrier function and controlling intestinal inflammation by activating aryl hydrocarbon receptor (AHR) (Scott et al. 2020; Pernomian et al. 2020). Recently, a tryptophan metabolite derived from *P. anaerobius* , trans‐3‐indoleacrylic acid (IDA), increased FSP1‐CoQH_2_ levels via the AHR‐aldehyde dehydrogenase 1 family member A3 (ALDH1A3) axis, thus contributing to ferroptosis resistance in CRC mouse models (Zhang, Kang, and Tang 2024; Cui et al. 2024) (Figure 3C).

## 5 Microbiome Influences on Ferroptosis in Disease

**FIGURE 4:**
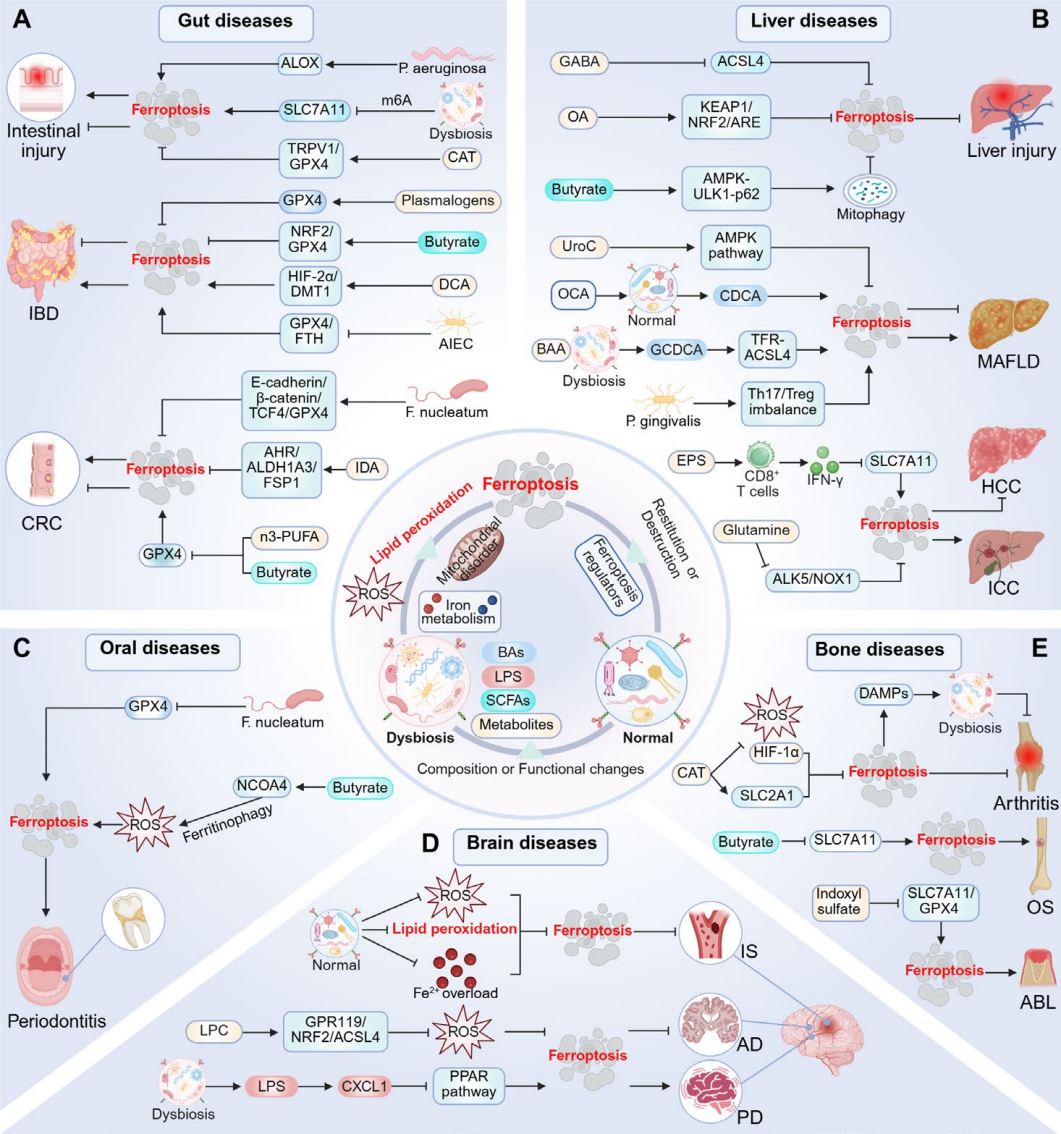
Crosstalk between the microbiome and ferroptosis in disease. The microbiome regulates ferroptosis in several ways to influence gut, liver, brain, oral, bone, and other diseases. ABL, alveolar bone loss; ACSL4, acyl‐CoA synthetase long‐chain family member 4; AD, Alzheimer's disease; AHR, aryl hydrocarbon receptor; AIEC, adherent‐invasive *E. coli* ; ALDH1A3, aldehyde dehydrogenase 1 family member A3; ALK5, activin receptor‐like kinase 5; ALOX, lipoxygenase; BAA, bromoacetic acid; BAs, bile acids; CAT, capsiate; CDCA, chenodeoxycholic acid; CRC, colorectal cancer; CXCL1, C‐X‐C motif chemokine ligand‐1; DAMPs, danger‐associated molecular patterns; DCA, deoxycholic acid; DMT1, divalent metal transporter 1; EPS, exopolysaccharides; FSP1, ferroptosis suppressor protein 1; GCDCA, glycochenodeoxycholate; GPX4, glutathione peroxidase 4; HCC, hepatocellular carcinoma; HIF‐1α, hypoxia‐inducible factor‐1α; HIF‐2α, hypoxia‐inducible factor‐2α; IBD, inflammatory bowel disease; ICC, intrahepatic cholangiocarcinoma; IDA, trans‐3‐indoleacrylic acid; IS, ischemic stroke; KEAP1, Kelch‐associated protein 1; LPC, lysophosphatidylcholine; LPS, lipopolysaccharide; MAFLD, metabolic dysfunction‐associated fatty liver disease; n‐3 PUFA, omega‐3 polyunsaturated fatty acids; NCOA4, nuclear receptor co‐activator 4; NOX1, NADPH oxidases 1; NRF2, nuclear factor erythroid 2‐related factor 2; NRF2, nuclear factor erythroid 2‐related factor 2; OA, oleanolic acid; OCA, obeticholic acid; OS, osteosarcoma; *P. gingivalis* , *Porphyromonas gingivalis* ; PD, Parkinson's disease; PPAR, peroxisome proliferator‐activated receptor; SCFAs, short‐chain fatty acids; SLC2A1, solute carrier family 2 member 1; TRPV1, transient receptor potential cation channel subfamily V member 1; UroC, urolithin C.

With increasing research, the regulatory role of the microbiome in ferroptosis is becoming increasingly clear. Not only does it play a role in gut diseases, but the microbiome can also participate in the progression of various non‐gut diseases (such as liver, brain, oral, and bone diseases) through the gut‐organ axis. In addition to the gut microbiome, crosstalk between the oral microbiome and ferroptosis has received considerable research attention (Figure 4). Notably, the interaction between the microbiome and ferroptosis in disease contexts has a dual role; that is, it both potentially exacerbates pathology and offers therapeutic avenues. For instance, in benign diseases, ferroptosis usually leads to disease progression, while in tumors, ferroptosis reverses chemotherapy resistance and promotes tumor cell death, providing new opportunities for cancer therapy. Therefore, it is highly necessary to identify specific microbial elements associated with different disease contexts.

### 5.1 Gut Diseases

#### 5.1.1 Microbiota Regulates Ferroptosis

Intestinal injury is closely associated with ferroptosis and caused by several factors, including environmental toxicants (Tang et al. 2023), ionizing radiation (Zhou et al. 2022), and intestinal IRI (Li et al. 2019). Fluoride is a common environmental toxicant (Stockbridge and Wackett 2024), with fluoride‐induced gut microbiome dysbiosis decreasing SLC7A11 expression via m6A modification, triggering ferroptosis in IECs, and causing IEC damage (Huang, Lin, et al. 2024). Total body irradiation (TBI) is another factor in gut microbiome dysbiosis and intestinal epithelial injury (Zhao et al. 2021). In a TBI environment, *P. aeruginosa* increases its intestinal colonization and catalyzes the peroxidation of arachidonic acid‐phosphatidylethanolamine (AA‐PE) to 15‐hydroperoxy‐AA‐PE via ALOX, resulting in IEC‐based ferroptosis (Dar et al. 2022). IBD, which includes Crohn's disease (CD) and ulcerative colitis (UC), is closely related to microbial metabolism (Shan et al. 2022). Notably, the gut microbiome not only influences IBD progression through metabolic pathways but also participates in the onset and development of the disease by regulating ferroptosis. Studies have shown that ferroptosis destroys intestinal barriers and is associated with dextran sodium sulfate (DSS)‐induced colitis (Wu et al. 2023). In CD, gut microbiome dysbiosis is characterized by reduced microbial diversity and the increased abundance of specific mucosal‐associated bacteria, particularly AIEC (de Souza et al. 2012). Notably, such colonization of AIEC contributes to the pathogenesis of CD through regulating ferroptosis; it exacerbates LPO and ferroptosis in IECs by reducing the levels of GPX4 and FTH, thereby inducing intestinal inflammation (Wen et al. 2023). The microbiome and ferroptosis are reportedly strongly involved in CRC pathogenesis (Permain et al. 2024). The oral microbiome has been shown to regulate ferroptosis in CRC via the gut‐oral axis. Ferroptosis is well known for its anti‐drug resistance to cancer therapy (Hassannia et al. 2019). *F. nucleatum* , a carcinogenic oral microbe, is believed to be involved in CRC initiation, progression, and chemotherapy resistance (Kong et al. 2023). Li, Wei, et al. (2024) reported that *F. nucleatum* inhibits ferroptosis through the E‐cadherin/β‐catenin/TCF4/GPX4 axis and promotes oxaliplatin resistance in CRC.

#### 5.1.2 Microbial Metabolites Regulate Ferroptosis

Different gut microbial metabolites exert different effects on ferroptosis and the above gut diseases. IRI, caused by oxidative stress in the ischemic intestines after blood supply restoration, is often accompanied by intestinal barrier destruction and microbiome changes (Chen, Han, et al. 2024). A previous study reported that CAT inhibited ferroptosis and reduced IRI‐mediated small intestinal mucosal damage by promoting TRPV1 and GPX4 expression (Deng et al. 2021). Obligate anaerobes, such as *Bifidobacterium longum* and *Clostridium butyricum* and their products, are potentially beneficial to the IBD colonic environment (Sharma et al. 2023; Wu et al. 2022). Plasmalogen, the most common ether phospholipid form in *Bifidobacterium longum* (Vítová et al. 2021), inhibits ferroptosis and reduces intestinal inflammation by increasing GPX4 levels but inhibiting COX‐2 expression (Liu et al. 2023). *Clostridium butyricum* ‐derived butyrate inhibited ferroptosis via NRF2/GPX4 signaling, improved intestinal barrier integrity, and effectively alleviated DSS‐induced colitis in mice (Chen, Qian, et al. 2024). In contrast, DCA potentially impairs the mucus barrier and aggravates UC progression (Liu et al. 2018). Long‐term high‐fat diets can increase DCA levels, which cause Fe^2+^ accumulation and ferroptosis in IECs by upregulating HIF‐2α and DMT1 expression and aggravating colitis (Wang, Chu, Dong, et al. 2024). A significant increase in *P. anaerobius* abundance was observed in the feces from CRC patients (Long et al. 2019). Its metabolite IDA inhibits ferroptosis through the AHR‐ALDH1A3‐FSP1 axis and promotes CRC progression (Cui et al. 2024). Additionally, the n‐3 PUFA and butyrate combination inhibits CRC development by weakening mitochondrial antioxidant defenses and promoting mitochondrial GPX4‐dependent ferroptosis (Chapkin et al. 2020).

### 5.2 Liver Diseases

Gut microbiome effects on liver diseases are mainly realized via the gut‐liver axis (Tilg et al. 2022; Aron‐Wisnewsky et al. 2020). As the role of ferroptosis in liver diseases becomes clearer, the interactions between the gut microbiome and ferroptosis are being uncovered. Notably, the novel oral‐gut‐liver axis links oral to liver diseases and provides strong support for oral pathogens in regulating hepatocyte ferroptosis (Albuquerque‐Souza and Sahingur 2022).

#### 5.2.1 Microbiota Regulates Ferroptosis

The gut microbiome can influence liver tumor progression by modulating ferroptosis. A recent study indicates that the gut microbiota inhibits ferroptosis in intrahepatic cholangiocarcinoma (ICC) cells by altering glutamine metabolism to inhibit the ALK5/NOX1 axis, which promotes ICC (Zhang, Zhou, Zhai, et al. 2024). Also, *Enterobacter faecium* induces CD8^+^ T cells to secrete IFN‐γ, which promotes ferroptosis in hepatocellular carcinoma (HCC) cells by down‐regulating SLC7A11, thus hindering disease progression (Yu, Lin, et al. 2024). In recent years, guidelines have recommended that NAFLD should be replaced with metabolic dysfunction‐associated fatty liver disease (MAFLD) (Rinella et al. 2023; Yang, Zhu, et al. 2024); therefore, MAFLD is used to describe NAFLD in this review. Accumulated studies now show that microbiome dysbiosis, one of the main MAFLD features (Saeed et al. 2024), influences MAFLD progression by modulating ferroptosis (Bu et al. 2024). Notably, oral microbes modulate hepatocellular ferroptosis, possibly via the oral‐gut‐liver axis (Albuquerque‐Souza and Sahingur 2022; Chen et al. 2023). In a study, oral *P. gingivalis* administration alters the gut microbiome and metabolites to induce a Th17/Treg imbalance and hepatocyte ferroptosis, thus causing MAFLD in mice (Yao et al. 2023). Interestingly, another study reported that the gut microbiome in mice orally administered *P. gingivalis* had colonized hepatocytes and induced ferroptosis, thereby exacerbating alcoholic liver disease (Yao et al. 2024).

#### 5.2.2 Microbial Metabolites Regulate Ferroptosis

In addition, microbial metabolites also affect MAFLD progression by regulating ferroptosis. The gut metabolite urolithin C inhibits ferroptosis via AMPK pathway activation and counteracts gut microbiome dysbiosis, showing an ameliorative potential for MAFLD (Xu et al. 2023). Obeticholic acid (OCA) increases chenodeoxycholic acid (CDCA) production by remodeling the gut microbiome; subsequently, CDCA promotes MAFLD fibrosis by inducing hepatocyte ferroptosis. This action was possibly related to OCA inducing LPO and impairing its antifibrotic effects (Zhuge et al. 2023). Additionally, environmental toxins induce gut microbiome dysbiosis and hepatocyte ferroptosis (Mu et al. 2022). Under bromoacetic acid exposure, the gut microbial metabolite glycochenodeoxycholate activates TFR‐ACSL4‐mediated ferroptosis to accelerate MAFLD (Liu, Gao, et al. 2022). IRI‐related ferroptosis is one major cause of liver injury (Liu et al. 2019; Ye et al. 2020; Jiao et al. 2020). A recent study reported that the microbial metabolite oleanolic acid inhibits hepatocyte ferroptosis via KEAP1/NRF2/ARE signaling and rescues severe IRI after liver transplantation (Pi et al. 2024). Acetaminophen (APAP)‐induced liver injury is the leading cause of acute liver failure (Reuben et al. 2016). Butyrate, a *Lachnospiraceae* metabolite, reportedly inhibits ferroptosis by activating AMPK‐ULK1‐p62 signaling and mitophagy, protecting mice from APAP‐induced acute liver injury (ALI) (Yang, Chang, et al. 2024).

### 5.3 Brain Diseases

The gut microbiome is involved in regulating oxidative stress in the CNS via the gut‐brain axis and affects brain neuron injury (Shandilya et al. 2022).

#### 5.3.1 Microbiota Regulates Ferroptosis

Ischemic stroke (IS) is a cerebrovascular disease with a significant global health burden, and its pathogenesis is associated with ferroptosis (Tuo et al. 2022). Currently, drug reperfusion or surgery is the major therapeutic strategy (Widimsky et al. 2023), but ischemia–reperfusion may induce ferroptosis by increasing oxidative stress, thus aggravating brain tissue injury (Cui et al. 2021). A recent animal study reported that changes in specific gut microbe abundance in mice significantly reduce oxidative stress and inhibit ferroptosis and alleviate neuronal damage (Wang, Zhang, et al. 2023). Moreover, restoring normal gut microbiota composition with fecal microbiota transplantation (FMT) effectively reduces LPO and iron overload levels, inhibiting ferroptosis and improving IS prognosis outcomes (Wei, Wang, et al. 2024). Parkinson's disease (PD) is mainly caused by α‐syn accumulation and DA neuron damage in the substantia nigra (SN) (Tansey et al. 2022). The α‐syn originates from the gut and enters the brain via the vagus nerve of the gut‐brain axis, where it is aggregated by activated microglia (Fang et al. 2024; Ebedes and Borlongan 2024). Notably, α‐syn is also a key factor inducing ferroptosis in neuronal cells (Dong‐Chen et al. 2023). Ma et al. (2024) found that the gut microbiota in rotenone‐treated mice were changed, manifested as enrichment of *Proteobacteria*, which raised the circulatory levels of pro‐inflammatory cytokines LPS and C‐X‐C motif chemokine ligand‐1 (CXCL1). Increased CXCL1 effectively induces microglial activation, α‐syn aggregation, iron accumulation, and ferroptosis in SN, thus causing neuroinflammation and dopaminergic neurodegeneration. Furthermore, they also demonstrated that supplementing linoleic acid to activate PPAR alleviated PD symptoms in CXCL1 mice. This indicates that modulation of gut microbiota reduces brain inflammation, oxidative stress, and neuronal ferroptosis in PD models (Ma et al. 2024).

#### 5.3.2 Microbial Metabolites Regulate Ferroptosis

The β‐amyloid (Aβ) deposition is a widely recognized pathological mechanism in Alzheimer's disease (AD) (Blennow et al. 2006). Evidence now suggests that GSH and GPX4 depletion, represented by ferroptosis, is closely related to the Aβ burden (Wang et al. 2022; Fan et al. 2024). Also, microglia M1 polarization caused by increased iron levels intensified brain neuroinflammation in AD patients (Alrouji et al. 2024). A recent study reported that the probiotic *Bacteroides ovatus* metabolite lysophosphatidylcholine inhibits ferroptosis by reducing intracellular ROS and LPO through the GPR119‐NRF2‐ACSL4 axis, thus reducing brain Aβ deposition and alleviating AD symptoms in mice (Zha et al. 2025).

### 5.4 Oral Diseases

The oral microbiome, the second largest microbiome in the body, directly regulates ferroptosis in oral diseases (Sedghi et al. 2021). Periodontitis is characterized by oral microbiome dysbiosis and immune inflammatory responses, which are caused by excessive *P. gingivalis* and *F. nucleatum* colonization (Teles et al. 2022). *P. gingivalis* releases inflammatory cytokines and triggers host immune inflammatory responses, thus promoting periodontitis and its pathological characteristics (Baker et al. 2024). Ferroptosis may be another contributing factor to periodontitis (Fu et al. 2023). An enhanced ferroptosis pathway was observed in patients with periodontitis and was possibly related to increased butyrate and decreased GSH and GPX4 levels (Ding et al. 2024). For example, *F. nucleatum* has been reported to induce ferroptosis in PDLSCs by regulating cellular iron metabolism, mitochondrial function, and GPX4 expression, thereby activating early‐stage inflammatory responses in hosts (Wang, Wang, et al. 2023). Unlike the anti‐inflammatory effects of butyrate at the gut level (Kang et al. 2023), butyrate secreted by periodontal pathogens disrupts iron homeostasis by promoting NCOA4‐mediated ferritinophagy, resulting in excessive ROS production and depleted GSH levels, which trigger ferroptosis in periodontal ligament fibroblasts and accelerate periodontitis (Zhao et al. 2020).

### 5.5 Bone and Joint Diseases

#### 5.5.1 Microbiota Regulates Ferroptosis

The gut‐bone axis allows the gut microbiome to influence bone diseases. Furthermore, the microbiome‐immunity axis has important roles in rheumatoid arthritis (RA) (Wang and Xu 2019). RA is an autoimmune disease that is activated by danger‐associated molecular patterns (DAMPs) in the innate immune system (Yasuda et al. 2024). P2X7 receptor (P2X7R) is a receptor on immune cells, inducing cellular inflammation and immunity activation (Di Virgilio et al. 2017). Notably, ferroptosis may alter the distribution and abundance of the gut microbiome and increase cell immunogenicity by activating DAMPs to activate P2X7R in a mouse model of RA (Ma et al. 2023). Equally, gut microbiome dysbiosis may cause immune dysfunction, which worsens joint destruction and inflammation and promotes RA progression (Ma et al. 2023).

#### 5.5.2 Microbial Metabolites Regulate Ferroptosis

Indoxyl sulfate is a gut toxin produced by tryptophan metabolism in the gut bacteria and induces IEC damage by promoting ROS production and interfering with mitophagy (Huang et al. 2020). The latest study reported that indoxyl sulfate activates AHR to inhibit osteoblast differentiation and reduce SLC7A11/GPX4 expression, which induces ferroptosis and aggravates alveolar bone loss (Chen et al. 2025). Solute carrier family 2 member 1 (SLC2A1), also known as GLUT1, is explicitly involved in ferroptosis, and its responses to hypoxia‐induced oxidative stress are mediated by HIF‐1α. Guan et al. (2023) observed that CAT shows good alleviating effects toward osteoarthritis; such remission effects are achieved by restraining ferroptosis, which is related to increased SLC2A1 expression, and inhibiting HIF‐1α signaling and oxidative stress levels. Additionally, butyrate (HDAC inhibitor) inhibits SLC7A11 transcription by upregulating ATF3 expression and promoting ferroptosis in osteosarcoma cells, thus alleviating osteosarcoma (Nie et al. 2023).

### 5.6 Other Diseases

In addition to the aforementioned microbiome sites, microbiome and ferroptosis crosstalk has been reported elsewhere in the body. For example, the microbial metabolite Urolithin A inhibited LPS‐induced acute lung injury and ferroptosis by upregulating the Keap1/NRF2/HO‐1 pathway (Lou et al. 2023). LPS induces ferroptosis‐mediated esophageal mucosal injury by increasing the expression of the special protein 1/ACSL4 and the accumulation of iron (Liu, Tang, et al. 2022). Probiotic *Prevotella histicola* reduces ferroptosis by inhibiting ACSL4 and activating the Xc^−^/GPX4 axis, thereby attenuating ethanol‐induced gastric mucosal lesions in mice (Peng, Lei, et al. 2024). Gut bacteria metabolite 3‐hydroxyphenylacetic acid inhibits ferroptosis and restores spermatogenesis in aged mice testicles (Teles et al. 2022). High‐fat diet‐induced gut microbiome dysbiosis increased circulating LPS level in mice, which promotes ferroptosis by decreasing GPX4 expression, thereby leading to testicular inflammation (Fu et al. 2023) and atrial inflammation (Ding et al. 2024). *Bacteroidaceae* mediates iron overload and lipid peroxidation to induce ferroptosis, thus promoting systemic inflammatory responses and benzene‐induced hematopoietic toxicity. Oral probiotics can reverse elevated *Bacteroidaceae* and ferroptosis, ultimately alleviating hematopoietic damage (Kang et al. 2023). These mechanisms are summarized (Table 1). However, crosstalk at these sites remains understudied. More evidence is required to explain these interactions.

**TABLE 1:** Microbiome influences on ferroptosis in disease.

Organs	Diseases	Microbiotas and metabolites	Relevant mechanisms	Result of ferroptosis	Progression of diseases	References
Gut	Intestinal injury	*Lactobacillus* and *Ileibacterium*	The m6A level of SLC7A11 mRNA is upregulated, which promotes YTHDF2 to bind to it and drive SLC7A11 mRNA degradation	Promote	Promote intestinal epithelial cell injury	(Huang, Lin, et al. 2024)
	Intestinal IRI	Capsiate	Activation of TRPV1 promotes GPX4 expression	Suppress	Suppress intestinal IRI	(Deng et al. 2021)
	IBD	Plasmalogens (anaerobes)	Regulates the expression of ferroptosis‐related proteins such as GPX4 and COX2	Suppress	Suppress colitis	(Liu et al. 2023)
		Butyrate	Upregulate the NRF2/GPX4 pathway	Suppress	Suppress colitis	(Chen, Qian, et al. 2024)
		Deoxycholic acid	Upregulate the HIF‐2α/DMT1 pathway	Promote	Strengthening of colitis	(Wang, Chu, Dong, et al. 2024)
		AIEC	Exacerbate lipid peroxidation by reducing GPX4 and FTH levels	Promote	Promote intestinal epithelial cell death	(Wen et al. 2023)
	CRC	IDA ( *P. anaerobius* )	Up‐regulate the AHR‐ALDH1A3‐FSP1 axis	Suppress	Promote tumorigenesis	(Cui et al. 2024)
		Butyrate	Downregulated GPX4 expression	Promote	Suppress tumor formation	(Chapkin et al. 2020)
		*F. nucleatum*	Up‐regulate the E‐cadherin/β‐catenin/TCF4/GPX4 axis	Suppress	Promote drug resistance	(Li, Wei, et al. 2024)
Liver	Liver IRI	Gamma‐aminobutyric acid	Reduces ACSL4, TFR1 and increases anti‐ferroptotic FTH1 levels	Suppress	Suppress liver IRI	(Wang, Liu, Huang, et al. 2024)
		Oleanolic acid	Upregulate the KEAP1/NRF2 pathway	Suppress	Alleviate liver IRI	(Pi et al. 2024)
	Acute liver injury	Butyrate (*Lachnospiracease*)	Activates the AMPK‐ULK1‐p62 pathway and mitophagy	Suppress	Alleviate acute liver injury	(Yang, Chang, et al. 2024)
	MAFLD	Urolithin C	Activates the AMPK pathway	Suppress	Suppress liver injury	(Xu et al. 2023)
		Chenodeoxycholic acid	Induces lipid peroxidation	Promote	Promote liver fibrosis	(Zhuge et al. 2023)
		Glycochenodeoxycholate	Activates TFR‐ACSL4	Promote	Promote hepatic steatosis and inflammation	(Liu, Gao, et al. 2022)
		*Porphyromonas gingivalis*	Th17/Treg imbalance	Promote	Induce the occurrence of NAFLD	(Yao et al. 2023)
	ALD	*Porphyromonas gingivalis*	Increased the expression of ACSL4 and decreased the expression of GPX4 and SLC7A11	Promote	Aggravate ALD	(Yao et al. 2024)
	ICC	Glutamine	Inhibits the ALK5/NOX1 axis	Suppress	Promote tumor growth	(Zhang, Zhou, Zhai, et al. 2024)
	HCC	EPS (*Enterobacter faecium*)	EPS promotes the secretion of IFN‐γ by CD8T cells, thereby down‐regulating SLC7A11	Promote	Enhance the efficacy of treatment	(Yu, Lin, et al. 2024)
Brain	IS	*Bacteroidaceae Enterobacteriaceae*	Reduce oxidative stress	Suppress	Suppress cerebral IRI	(Wang, Zhang, et al. 2023)
		Restoring normal gut microbiota composition	Reduce the levels of lipid peroxidation and iron overload	Suppress	Ameliorate IS	(Wei, Wang, et al. 2024)
	PD	*Enterobacterium* (*Proteobacteria*)	Elevated CXCL1 causes PPAR pathway inhibition, promoting microglia activation, α‐syn aggregation, and iron accumulation	Promote	Promote dopaminergic neurodegeneration in PD	(Ma et al. 2024)
	AD	*Lysophosphatidylcholine*	Reduces ROS and lipid peroxidation through the GPR119‐NRF2‐ACSL4 axis	Suppress	Ameliorate cognitive impairment	(Zha et al. 2025)
Oral	Periodontitis	Butyrate (Periodontal pathogens)	Promotes NCOA4‐mediated ferritinophagy, causing excessive ROS and depletion of GSH	Promote	Promote periodontitis development	(Zhao et al. 2020)
Bone	Alveolar bone loss	Indoxyl sulfate	Activates AHR to inhibit osteoblast differentiation and reduce SLC7A11/GPX4 expression	Promote	Promote alveolar bone deterioration	(Chen et al. 2025)
	Osteoarthritis	Capsiate	Inhibition of HIF‐1α and activation of SLC2A1	Suppress	Improve osteoarthritis	(Guan et al. 2023)
	Osteosarcoma	Butyrate	Inhibits SLC7A11 expression through ATF3	Promote	Suppress tumor growth	(Nie et al. 2023)
Lung	Lung cancer	Butyrate (Gut microbiota)	Inhibits SLC7A11 expression through ATF3	Promote	Suppress cancer cell survival	(Bi et al. 2024)
	Acute lung injury	Urolithin A	Activates the Keap1‐NRF2/HO‐1 signaling pathway	Suppress	Suppress acute lung injury	(Lou et al. 2023)
Esophagus	RE	LPS (Gram‐negative bacteria)	Promotes ACSL4 by elevating the expression of Sp1 through the Caspase11/GSDMD pathway	Promote	Promote esophageal mucosal damage	(Liu, Tang, et al. 2022)
Stomach	GC	*Helicobacter pylori* with CagA	Activates the MEK/ERK/SRF pathway	Promote	Increase drug sensitivity	(Peng, Lei, et al. 2024)
			Reduced NOX4 and ACSL4 expression, upregulated GPX4 expression	Suppress	Promote tumor growth	(Lu et al. 2024)
Testicle	Oligospermia	3‐HPAA	Reduce oxidative stress and elevate the expression of GPX4	Suppress	Alleviated spermatogenic dysfunction	(Jin et al. 2023)
	Testicular injury	LPS	The expression of NRF2 was downregulated	Promote	Induce testicular inflammation	(Zhang, Chen, and Li 2024)
Heart	AF	LPS (*Desulfovibrionaceae*)	Activates ferroptosis and the TLR4/NF‐κB/NLRP3 pathway	Promote	Increase susceptibility to AF	(Kong et al. 2022)
Blood system	AML	Propionate	Caused excessive ROS to induce mitochondrial damage and fission and mitophagy; increased ACSL4	Promote	Increase the sensitivity to immunotherapy	(Wei, Liu, et al. 2024)
	Hematopoietic toxicity	*Bacteroidaceae*	Mediated systemic inflammatory responses, iron overload, and lipid peroxidation	Promote	Increase hematopoietic toxicity	(Zhang, Kang, et al. 2023)

Abbreviations: 3‐HPAA, 3‐hydroxyphenylacetic acid; ACSL4, acyl‐CoA synthetase long‐chain family member 4; AF, atrial fibrillation; AHR, aryl hydrocarbon receptor; AIEC, adherent‐invasive *E. coli* ; ALD, alcoholic liver disease; ALDH1A3, aldehyde dehydrogenase 1 family member A3; ALK5, activin receptor‐like kinase 5; AML, acute myeloid leukemia; ARA‐PEs, phosphatidylethanolamine‐anchored arachidonic acids; ATF3, transcription factor 3; COX2, cyclooxygenase 2; CRC, colorectal cancer; CXCL1, C‐X‐C motif chemokine ligand‐1; CYP2E1, cytochrome P450‐2E1; DMT1, divalent metal transporter 1; EPS, exopolysaccharides; FSP1, ferroptosis suppressor protein 1; FTH, ferritin heavy chain; GC, gastric cancer; GPX4, glutathione peroxidase 4; GSH, glutathione; HCC, hepatocellular carcinoma; HIF‐2α, hypoxia‐inducible factor‐2α; IBD, inflammatory bowel disease; ICC, intrahepatic cholangiocarcinoma; IDA, trans‐3‐indoleacrylic acid; IRI, ischemia reperfusion injury; IS, ischemic stroke; KEAP1, Kelch‐associated protein 1; LPS, lipopolysaccharide; LPS, lipopolysaccharide; m6A, N6‐methyladenosine; MAFLD, metabolic dysfunction‐associated fatty liver disease; NASH, nonalcoholic steatohepatitis; NCOA4, nuclear receptor co‐activator 4; NOX1, NADPH oxidases 1; NRF2, nuclear factor erythroid 2‐related factor 2; PD, Parkinson's disease; PPAR, peroxisome proliferator‐activated receptor; PUFA, polyunsaturated fatty acid; RE, reflux esophagitis; ROS, reactive oxygen; SLC7A11, solute carrier family 7 member 11; Sp1, special protein 1; SREBP1, sterol‐regulatory element binding protein 1; TCF4, transcription factor; TFR, transferrin receptor; TLR4, toll‐like receptor‐4; TRPV1, transient receptor potential cation channel subfamily V member 1; YTHDF2, YTH domain‐containing family protein 2.

## 6 Disease Treatments

In recent years, considering the critical roles of the microbiome and ferroptosis in disease progression, therapeutic strategies targeting their crosstalk have become increasingly common in the disease treatment field. Therefore, we conclude this review with current and major prevention and treatment interventions targeting this crosstalk (Table 2).

**TABLE 2:** Therapeutic interventions targeting crosstalk between the microbiome and ferroptosis in disease.

Type	Names	Model	Related targets	Mechanism action	Diseases	References
FMT	Fecal bacterial suspension	Rats with MCAO	Restores normal gut microbiota composition	Reduces cerebral infarction volume and ferroptosis after stroke	IS	(Wei, Wang, et al. 2024)
	OFG or OPG	APAP‐induced liver injury in mice	↑ *Lachnospiraceae* and butyrate	Changes the gut microbiome composition	ALI	(Yang, Chang, et al. 2024)
	Fecal microbiota gavage	Mice and GC‐2 spd cells	↑ 3‐hydroxyphenylacetic acid	Upregulates GPX4 to decrease oxidative stress	Oligospermia	(Jin et al. 2023)
Probiotics	*L. lactis* MG1363‐pMG36e‐GLP‐1	MTPT‐induced PD mice	↑ *Akkermansia*, *Oscillospira*, and *Sutterella*	Remodels gut microbiome homeostasis and decreases oxidative stress	PD	(Yue et al. 2022)
	LGG	DSS‐ or WAI‐induced colitis mice	↑ *Lactobacillus*, *Blautia*, and *Akkermansia* ↓ pathogenic *E. coli* and *Bacteroides*	Produces SCFAs and increases GPX4 to clear ROS	Colitis	(Sun et al. 2024)
	Bifico	Benzene‐induced hematopoietic toxicity in mice	↓ Bacteroidaceae	Inhibits gut ferroptosis and Th2‐type systemic inflammation	Hematopoietic toxicity	(Zhang, Kang, et al. 2023)
	*Bifidobacterium bifidum* BGN4	PA‐induced‐MAFLD LO2 cells	↓ CYP2E1	Decreases oxidative stress	MAFLD	(Bu et al. 2024)
	*Clostridium butyricum*	KPC1199 cells mice	↑ Butyrate	Maintains gut homeostasis	PDAC	(Yang, Zhang, et al. 2023)
	*P. histicola*	Ethanol‐induced EGML mice	Consolidate the gastric microbiota	Inhibits ACSL4 and activates the Xc^−^/GPX4 axis	EGML	(Wang, Wu, et al. 2023)
Prebiotics	TA 2–1	DSS‐induced UC mice	↑ *Akkermansia, Adlercreutzia*, and *Lactobacillus*	Increased amino acid levels to promote Xc^−^‐GSH‐GPX4 defense	UC	(Peng, Wang, et al. 2024)
	L‐Fucose	OTA‐induced ovarian damage in mice	↓ *Sutterella*	Counteracts microbial dysbiosis and inflammatory/ROS response	Ovarian damage	(Wang, Song, Cai, et al. 2024)
	EPS	Hepa1‐6 cell mice	↑ *Enterobacter faecium*	Promotes the secretion of IFN‐γ^+^ by CD8^+^ T cells to enhance sorafenib activity, increasing ROS	HCC	(Yu, Lin, et al. 2024)
TCM	JFG	CFA‐induced RA rat	↑ α diversity and SCFAs	Activates AMPK to inhibit lipid oxidative stress	RA	(Wang, Pan, et al. 2025)
	Berberine	Cerebral ischemia–reperfusion injury mice	↑ Bacteroidaceae and *Enterobacteriaceae* ↓ *Muribaculaceae*, *Erysipelotrichaceae*, *Helicobacteraceae*, *Streptococcaceae*, and *Tannerellaceae*	Produces SCFAs and increases GPX1 to decrease lipid peroxidation and oxidative stress	IS	(Wang, Zhang, et al. 2023)
	SHTB	MCAO/R rats	↑ *Lactobacillus* ↓ *Escherichia‐Shigella*	Increase SCFAs to induce PPARγ and reduce lipid oxidative stress	IS	(Wei et al. 2025)
	SDG	TMAO‐induced NRK‐52E cells and adenine‐induced CKD rats	↓ *Muribaculaceae*, *Bacteroides*, and *Ruminococcaceae_UCG‐010*	Decreases TMAO to reduce ROS	CKD	(Ge et al. 2024)
	Nobiletin	CLP‐induced SALI mice	↑ *Ligilactobacillus*, *Akkermansia*, and *Lactobacillus* ↓ *Dubosiella* and *Bacteroides*	Increase NRF2‐GPX4 axis	SALI	(Huang et al. 2023)

Abbreviations: APAP, acetaminophen; Bifico, *Bifidobacterium longum* , *Lactobacillus acidophilus* , and *Enterococcus faecalis* ; CFA, complete Freud's adjuvant; CKD, chronic kidney disease; CLP, cecal ligation and puncture; DSS, dextran sodium sulfate; EGML, ethanol‐induced gastric mucosal lesions; FMT, fecal microbiota transplant; JFG, Jingfang Granule; *L. lactis* , *Lactococcus lactis* ; LGG, *Lactobacillus rhamnosus* GG; MCAO, middle cerebral artery occlusion; MCAO/R, middle cerebral artery occlusion/reperfusion; MPTP, 1‐methyl‐4‐phenyl‐1,2,3,6‐tetrahydropyridine; OFG, oral fecal gavage; OPG, pasteurized fecal gavage; *P. histicola* , *Prevotella histicola* ; PA, palmitic acid; PDAC, pancreatic ductal adenocarcinoma; SALI, sepsis‐associated acute liver injury; SDG, suyin detoxification granule; SHTB, Shouhui Tongbian capsules; TCM, traditional Chinese medicine; TMAO, trimethylamine N‐oxide; WAI, whole‐abdominal irradiation.

### 6.1 Fecal Microbiota Transplantation

FMT is a promising treatment for disorders associated with gut microbiome dysbiosis, as it restores healthy gut microbiome diversity by transferring feces from healthy donors to recipients (Porcari et al. 2023). The evidence now shows that FMT generates satisfactory results in treating several diseases, including recurrent *Clostridioides difficile* infections (Kao et al. 2017), IBD (Lopetuso et al. 2023), and cancers (Yu, Feng, et al. 2024; Wang, Fang, et al. 2025). FMT improves intestinal inflammatory status, reduces oxidative stress, and restores intestinal barrier function by increasing beneficial microbes and reducing potentially pathogenic bacteria (Zu et al. 2024), which in turn reduces ferroptosis risks. For example, FMT reversed gut microbiota dysbiosis, reduced ferroptosis after IS, and relieved cerebral infarction symptoms (Wei, Wang, et al. 2024). However, limitations can occur in terms of patient acceptability and adherence to FMT, thus restraining its application to clinical practice. However, oral fecal gavage (OFG) or pasteurized fecal gavage (OPG) are more acceptable approaches for patients, while the efficacy is comparable to FMT administration by colonoscopy (Kao et al. 2017; Vaughn et al. 2023). The study authors reported that OFG or OPG improved ALI prognosis outcomes by altering gut microbiome structures and inhibiting ferroptosis (Yang, Chang, et al. 2024). Additionally, fecal microbiota component (mainly bacteria or the virome) transplantation, accompanied by higher patient acceptability when compared with crude FMT, may represent improved trends for the next generation of FMT (Yu, Wang, and Zhang 2023).

### 6.2 Probiotics and Prebiotics

Probiotics are living microbes that, when administered in sufficient quantities, provide health benefits to hosts (Fentie et al. 2024). Increasing evidence now shows that short‐term probiotic gut colonization alleviates gut microbiome dysbiosis and inhibits intracellular iron overload and LPO by improving intestinal barrier function, SCFA production, and reducing intestinal ROS levels to slow disease progression (Cao et al. 2023; Verstraeten et al. 2024). *Lactobacillus* and *Bifidobacterium* are the most common probiotics. *Lactobacillus* produces GSH and many different antioxidant enzymes (e.g., SOD) to reduce oxidative stress (Liu, Yue, et al. 2024). For instance, *L. lactis* MG1363‐PMGP36E‐GLP‐1 exerted neurotrophic effects by activating the Keap1/NRF2/GPX4 pathway to inhibit ferroptosis (Yue et al. 2022). Additionally, *Lactobacillus rhamnosus* GG improves colitis in mice by synthesizing SOD and SCFAs to inhibit ferroptosis (Sun et al. 2024). Moreover, Bifico (which contains *Bifidobacterium longum* , *Lactobacillus acidophilus* , and *Enterococcus faecalis* ) alleviates benzene‐induced hematopoietic toxicity by inhibiting *Bacteroidaceae*‐mediated ferroptosis (Zhang, Kang, et al. 2023).

Prebiotics are organic food substances that are not digested, are absorbed by hosts, and selectively support beneficial microbe growth and metabolism (e.g., *Bifidobacterium*, *Lactobacillus*, and *Fecalibacterium*) to improve host health (Sanders et al. 2019). Since the main role of prebiotics is to play a permissibility role on beneficial microbiomes in the gut, their efficacy against disease is inextricably linked to probiotic function (Quigley 2019). Studies have shown that TA 2–1 (Peng, Wang, et al. 2024) and L‐fucose (Wang, Song, Cai, et al. 2024) enhance intestinal barrier integrity by increasing intestinal probiotic proliferation, supporting IEC development, inhibiting ferroptosis in cells, and relieving UC and ovarian damage. In contrast, *E. faecium* exopolysaccharides synergistically induce ferroptosis in HCC cells with sorafenib by promoting IFN‐γ^+^ secretion from CD8^+^ T cells and reducing SLC7A11 levels (Yu, Lin, et al. 2024). Such is related to the anti‐resistance properties of ferroptosis in tumor treatment.

### 6.3 Traditional Chinese Medicine (TCM)

There is growing evidence that TCM effectively influences disease progression via the microbiome to regulate ferroptosis. Accordingly, Jingfang granules improve fatty acid metabolism disorders by regulating the gut microbiome and SCFA production to activate AMPK, inhibiting ferroptosis caused by lipid oxidative stress, and attenuating RA (Wang, Pan, et al. 2025). Berberine (Wang, Zhang, et al. 2023) and Shouhui Tongbian capsules (Wei et al. 2025) inhibit LPO and oxidative stress by increasing SCFA levels and preventing ferroptosis, thus improving IS symptoms. Suyin detoxification granules reduce ROS levels via the gut microbiome to reduce trimethylamine N‐oxide levels, thus suppressing renal tubular ferroptosis and preventing chronic kidney disease progression (Ge et al. 2024). Furthermore, nobiletin decayed ferroptosis via the gut microbiome to activate the NRF2‐GPX4 pathway and then mitigate sepsis‐associated ALI (Huang et al. 2023). This evidence underscores TCM as a promising treatment option for different diseases.

## 7 Conclusions and Prospects

Ferroptosis represents an imbalance in intracellular redox reactions and is a form of programmed cell death resulting from iron‐dependent LPO due to a ruptured cytoplasmic membrane. Although ferroptosis usually heralds tissue damage and necroinflammation, it has received considerable research attention in precision tumor therapy and as a novel strategy for combating therapeutic resistance. It is worth noting that there are physiological thresholds for the iron ratio, lipid oxidation, and total ROS levels in healthy cells. Once these thresholds are exceeded, the cells become more sensitive to ferroptosis. Using ferroptosis detection methods, such as the FerroOrange fluorescent probe, ferrozine, Prussian blue staining, the DCFH‐DA fluorescent probe, and C11‐BODIPY, it can be observed that when iron death occurs, the iron ratio in cells increases, and the fluorescence intensity of lipid oxidation and total ROS levels significantly enhances. Additionally, since ferroptosis shares similarities with other forms of cell death, combined detection of multiple markers (e.g., Fe^2+^+lipid peroxidation+mitochondrial morphological changes) can effectively help researchers confidently determine that ferroptosis is the primary contributor to cell death rather than other forms of cell death (Zeng et al. 2023).

It is now accepted that microbiome dysbiosis facilitates disease onset and progression, which is related to the regulation of the host's physiological processes, such as metabolism, redox reactions, and the immune system. Iron is an essential micronutrient for normal host physiological and microbiome metabolism. The cooperation and competition between the host and the microbiome for iron uptake have provided a theoretical basis for the microbiome‐mediated regulation of iron‐related diseases (Noordine et al. 2024; Das et al. 2020). Indeed, increasing evidence now suggests that toxic pathogen metabolites can induce ferroptosis and influence disease progression, whereas probiotics secrete beneficial metabolites that inhibit ferroptosis and alleviate disease symptoms. However, this evidence is not enough to fully elucidate the complex relationships between the microbiome and ferroptosis in disease. Most studies have posited that ferroptosis is regulated by the microbiome, but notably, the microbiome and ferroptosis may be in a mutual regulatory relationship. Some evidence now suggests that ferroptosis may, in turn, influence disease onset and progression by modulating the ecological balance of the gut microbiome. For example, triggering ferroptosis with a ferroptosis inducer (erastin) exacerbates gut microbiome dysbiosis in collagen‐induced arthritis mice (Ma et al. 2023; Zhu et al. 2025). Nevertheless, this is insufficient for a comprehensive and detailed discussion of the specific regulatory relationships between the microbiome and ferroptosis. More research is required to elucidate the sequential and causal relationships between microbiome dysbiosis and ferroptosis in disease.

Therapies targeting the crosstalk between microbiome and ferroptosis hold significant clinical value. Specifically, FMT, probiotics, prebiotics, and TCM have shown great potential in disease management by influencing ferroptosis primarily by modulating microbiome composition. Compared with probiotics and prebiotics, which only colonize the gut temporarily, FMT offers considerable therapeutic advantages due to its sustained effects. Furthermore, unlike antibiotics that disrupt gut microbiome balance and potentially increase drug resistance, FMT gently restores microbial homeostasis. However, poor accessibility and patient acceptability remain major limitations of FMT. Additionally, the crosstalk between the gut microbiome and ferroptosis may also provide a potential target for glucose‐lowering drugs to fulfill their therapeutic potential. For instance, metformin alleviates hepatic IRI symptoms by inhibiting ferroptosis through gut microbiome remodeling (Wang, Liu, Huang, et al. 2024). Furthermore, drugs such as ferrostatin‐1 (Wang, Li, et al. 2023), deferasirox (Wu et al. 2023), and L‐citrulline (Zhao et al. 2024) can reshape gut microbiome homeostasis while inhibiting ferroptosis, thus mitigating disease progression. However, the exact relationship between ferroptosis inhibition and reshaping microbiome homeostasis has not yet been elucidated. This may be related to the fact that ferroptosis has not been shown to definitively regulate the microbiome.

In conclusion, crosstalk between the microbiome and ferroptosis across different diseases is highly complex. When compared with its regulation of gut diseases, gut microbiome influences on liver, brain, oral, bone, and other parenteral diseases depend on gut‐organ axes. The oral microbiome influences disease progression by regulating ferroptosis. This novel finding will undoubtedly deepen our understanding of the crosstalk between the microbiome and ferroptosis. Future studies should explore this crosstalk at other body sites. Notably, human cells are more vulnerable to iron overload compared to rodent cells, which is related to their lower antioxidant defense efficiency, higher irreversible damage, and weaker response to repair pathways (Zheng et al. 2018; Ma et al. 2021; Du et al. 2025). Therefore, a cautious attitude still needs to be maintained regarding the extrapolation of translational research on mouse data. To fully exploit the therapeutic potential of the microbiome and ferroptosis, more comprehensive basic, preclinical, and clinical studies are necessary. Continued foundational and clinical research in this field may facilitate the translation from mechanistic understanding to clinical therapies in the future, though a cautiously optimistic approach remains essential at present.

## Author Contributions

D.‐Q.D. conceived the review. C.‐D.Z. provided guidance on the writing of the manuscript. Y.‐S.L. provided guidance on drawing figures. J.‐K.Z. provided guidance on the revision and correction of the manuscript. S.‐Q.D. was a major contributor to writing the manuscript. Y.‐L. organized the tables and was responsible for some of the writing. Z.‐M.Z. was responsible for the creation and modification of the figures. X.‐Y.L. collected the literature. J.‐X.L. collated the literature. All authors contributed to the discussion and revision and the final version.

## Ethics Statement

The authors have nothing to report.

## Consent

The authors have nothing to report.

## Conflicts of Interest

The authors declare no conflicts of interest.

## Data Availability

Data sharing not applicable to this article as no datasets were generated or analysed during the current study.
